# Microneedle‐Integrated Device for Transdermal Sampling and Analyses of Targeted Biomarkers

**DOI:** 10.1002/smsc.202200087

**Published:** 2023-04-22

**Authors:** Shubhangi Shukla, Sina Azizi Machekposhti, Naveen Joshi, Pratik Joshi, Roger J. Narayan

**Affiliations:** ^1^ Joint Department of Biomedical Engineering North Carolina State University Raleigh NC 27695-7907 USA; ^2^ Department of Materials Science and Engineering North Carolina State University Raleigh NC 27695-7907 USA

**Keywords:** 3D printing, diffusion, glucose monitoring, interstitial fluids, microneedle arrays, transdermal

## Abstract

Currently available point‐of‐care systems for body fluid collection exhibit poor integration with sensors. Herein, the design of a disposable device for interstitial fluid (ISF) extraction as well as glucose, lactate, and potassium ion (K^+^) monitoring is reported on. It is minimally invasive and appropriate for single use, minimizing the risk of infection to the user. This microscale device contains a 3D‐printed cap‐like structure with a four‐by‐four microneedle (MN) array, bioreceptor‐modified carbon fiber (CF)‐sensing surface, and negative pressure convection technology. These features are incorporated within a compact, self‐contained, and manually operated microscale device, which is capable of withdrawing ≈3.0 μL of ISF from the skin. MN arrays applied with an upward driving force may increase the ISF flow rate. Moreover, functionalized CF working electrodes (WE_1_, WE_2_, WE_3_) are shown to selectively detect lactate, glucose, and K^+^ with high sensitivities of 0.258, 0.549, and 0.657 μA μm ^−1^ cm^−2^ and low detection limits of 0.01, 0.080, 0.05 μm, respectively. Ex vivo testing on porcine skin is used to detect the ISF levels of the biomarkers. The microscale device can be a replacement for current point‐of‐care diagnostic approaches.

## Graphical abstract

This manuscript describes a minimally invasive microneedle cap‐integrated interstitial fluid collection and evaluation device with flexible carbon fiber‐based bioreceptor‐modified sensor interfaces. This single‐use platform is designed to assess glucose, lactate, and K^+^ levels via electrochemical measurements. The biosensor is shown to be highly sensitive and selective for detecting the analytes at physiologically‐relevant levels.

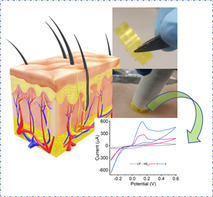

## 1 Introduction

Noninvasive and minimally invasive biomarker sampling from body fluids has the potential to revolutionize personalized medicine.^[^ ^1^, ^2^ ^]^ Biological fluids such as interstitial fluid (ISF) have been shown to possess important biological molecules such as proteins, peptides, metabolites (e.g., glucose, lactate, and alcohol), salts, and nucleic acids in close relation to the levels found in systemic circulatory fluids. The dermoepidermal junction is considered to be an appropriate site for painless ISF extraction (e.g., for the analysis of glucose, IgGs, lactate, and sodium–potassium equilibrium levels).^[^ ^1^ ^]^ Recent efforts on minimally invasive wearable sensors for biomarker detection involve the optical, chemical, and/or electrochemical detection of biomarker levels in serum, ISF, and sebaceous secretions. Microneedle (MN) arrays, which are microscale hypodermic needles that may be made of metallic or nonmetallic materials, have attracted attention from the scientific community for ISF acquisition and handling. These devices can generate micrometer‐scale pathways in the stratum corneum, the topmost layer of the epidermis, for body fluid extraction.^[^ ^1^, ^3^, ^4^, ^5^ ^]^

Numerous studies reported on the design of MN patches for transdermal drug delivery and biosensing. For example, Chua et al. described the skin penetration and ISF collection parameters that were associated with various shapes of MN arrays.^[^ ^6^ ^]^ Prototypes containing a tapered MN array showed a higher current signal (400 nA) than prototypes containing a straight MN array (250 nA). However, the sensitivity of this approach for glucose sensing was noted to be low. Zimmermann et al. employed a diffusion approach for glucose monitoring via MN patches; however, this approach involved elevated temperatures.^[^ ^7^ ^]^ In another approach, Wang et al. developed a glucose detection system that involved injecting MNs to a depth of 700 μm–1.5 mm; however, this system utilized vacuum suction (pressure=200–500 mm Hg) for 2–10 min using bulky equipment.^[^ ^8^ ^]^ Wang et al. reported on the development of a sensor‐enclosing device (SED) for ISF collection via passive diffusion; this device contained an in‐house developed membrane biosensor (MB) and MN array.^[^ ^9^ ^]^ Samant et al. examined a series of ISF collection mechanisms that involved diffusion, vacuum convection, capillary action, osmosis (involving a hydrogel), and hollow MN arrays. ISF flow rates varied according to the presence and absence of convective forces; the efficiency of the evaluated techniques was ranked in the following order: diffusion < capillary action < osmosis < applied pressure/suction.^[^ ^1^ ^]^ However, their model of vacuum‐driven systems was complex and cumbersome; moreover, ISF isolation rates were limited by variable tissue hydration properties. Due to the limitations of currently available ISF sampling procedures, there is a need for a straightforward, safe, standardized, and painless approach that can be utilized both within and outside traditional healthcare settings.

Most MN‐based direct ISF measurement systems, including the aforementioned systems, have been restricted to the evaluation of a single biomarker. These continuous glucose monitoring systems also did not exhibit good integration between ISF collection and sensing components.^[^ ^5^, ^6^ ^]^ Some wearable or on‐body sensors developed so to this point have demonstrated the capability for detecting multiple analytes.^[^ ^8^ ^]^ However, these arrangements usually involve sweat sensors,^[^ ^10^, ^11^, ^12^ ^]^ complex electronic interfaces, and metabolically unrelated analytes (e.g., ethanol, cholesterol, dopamine, and glucose).

Analytes such as glucose, potassium ion, and lactate are important for diagnosing several chronic medical conditions. Therefore, detecting the levels of these analytes in body fluids can offer improved insight into a given individual's physiological state.^[^ ^10^ ^]^ Here, we report on the development of a MN array‐based point‐of‐care microscale device for ISF extraction and analyte monitoring. This device provides the analysis of three biomarkers, namely glucose, lactate, and potassium ion levels. The MN arrays utilized the pressure‐driven convection technique to withdraw ISF. This facile method enabled the acquisition of a sufficient ISF volume (≈3.0 μL) for downstream analyses. The multiplexed microscale device includes a 3D‐printed four‐by‐four circular MN array cap at the one end, a thin cotton pad, asyringe‐like polydimethylsiloxane (PDMS) mold, a gel electrolyte slab with microchannels, as well as a three‐electrode system containing a reference electrode, a counter electrode, and three‐carbon fiber (CF)‐based working electrodes (WEs). MNs were designed with similar features as the 2D MN arrays previously fabricated by Mukherjee et al.; in this study, a suction‐induced negative pressure feature was incorporated in the microscale device.^[^ ^13^ ^]^ The oblique structure of MNs enabled significant stretching of the epidermal layer at the MN tip to prevent skin from folding in proximity to the MN tip; this attribute enhanced the skin penetration process. The ISF that is released from the skin is absorbed into the thin flat cotton swab () and through fine microchannels in the agar gel with redox mediator; it is then made available to the surface of fiber WEs. CF, being conducting, flexible, exhibiting a high specific surface area, and exhibiting highly selective behavior, acted as an ideal biosensing material.

The CF‐based electrodes were coated with siloxane to obtain a flat surface for maximum electrocatalytic efficiency. The integrated system, which contained three fiber electrodes with distinct components, was assembled after modification with appropriate receptors. Owing to the excellent adsorption properties of activated CFs,^[^ ^14^, ^15^, ^16^ ^]^ they were functionalized with Pd@Au core shell nanoparticle dispersions along with specific catalytic bioreceptors glucose oxidase (GO_*x*_), lactate oxidase (LO_*x*_), and 4‐aminobenzo‐18‐Crown‐6‐ether (AOCE‐6). This integrated portable microscale device enabled the parallel electrochemical detection of three clinically relevant analytes.^[^ ^15^, ^16^, ^17^ ^]^ The MN–CF electrochemical interface was used to obtain data from ISF and in vitro solutions. The microscale device is made from cost‐effective CF components; as such, the microscale device can serve as a single‐use disposable sensor. In addition, a 2D symmetric model of the electroactive surface was developed using COMSOL Multiphysics 6.0 software to visualize the molecular transport. Appropriate external environment and boundary conditions were used to study the free diffusion of analytes according to analytical equations.^[^ ^18^ ^]^

## 2 Results and Discussion

### 2.1 Designing MN‐Based Biosensor Device

**Figure 1:**
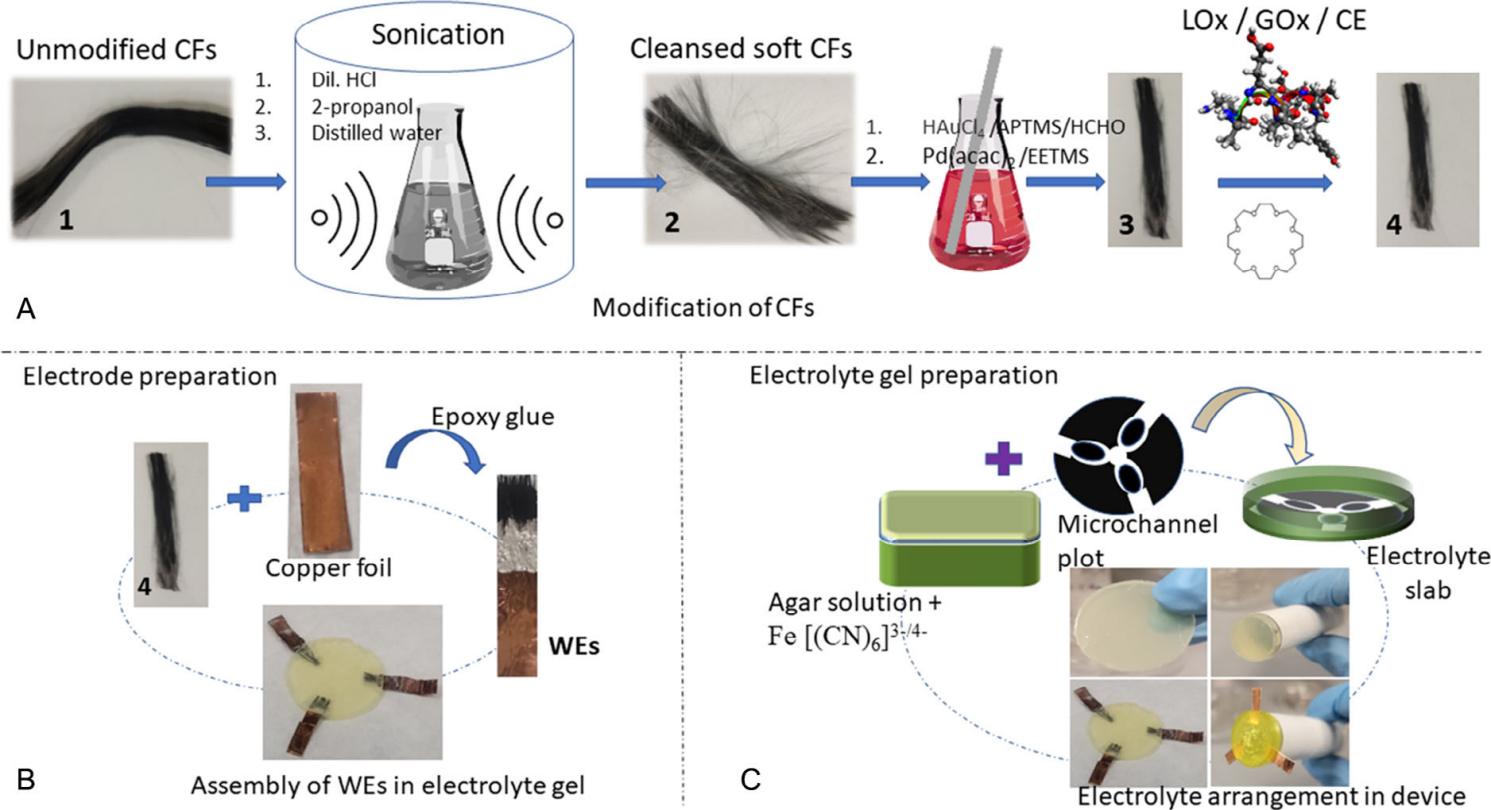
Schematic illustration of CF WEs fabrication. A) Sonication treatment and functionalization of CFs with LO_*x*_, GO_*x*_, and AOCE‐6 to make WE_1_, WE_2,_ and WE_3_, B) Attachment of CFs with copper foil using silver epoxy resin, and C) preparation of electrolyte gel as well as assembly of three WEs into gel slab and device. Symbols were defined as: CFs, carbon fibers; HCl, hydrochloric acid; HAuCl_4_, tetrachloroauric acid; APTMS, 3‐aminopropyl trimethyl siloxane; HCHO, formaldehyde; Pd(acac)_2,_ palladium acetylacetonate; EETMS, 2 ‐(3.4 epoxycyclohexyl) ethyltrimethoxy silane; LO_*x*_, lactate oxidase; GO_*x*_, glucose oxidase; CE, crown ether; Wes, working electrodes; Fe [(CN)_6_]^3−/4−^, potassium ferricyanide.

**Figure 2:**
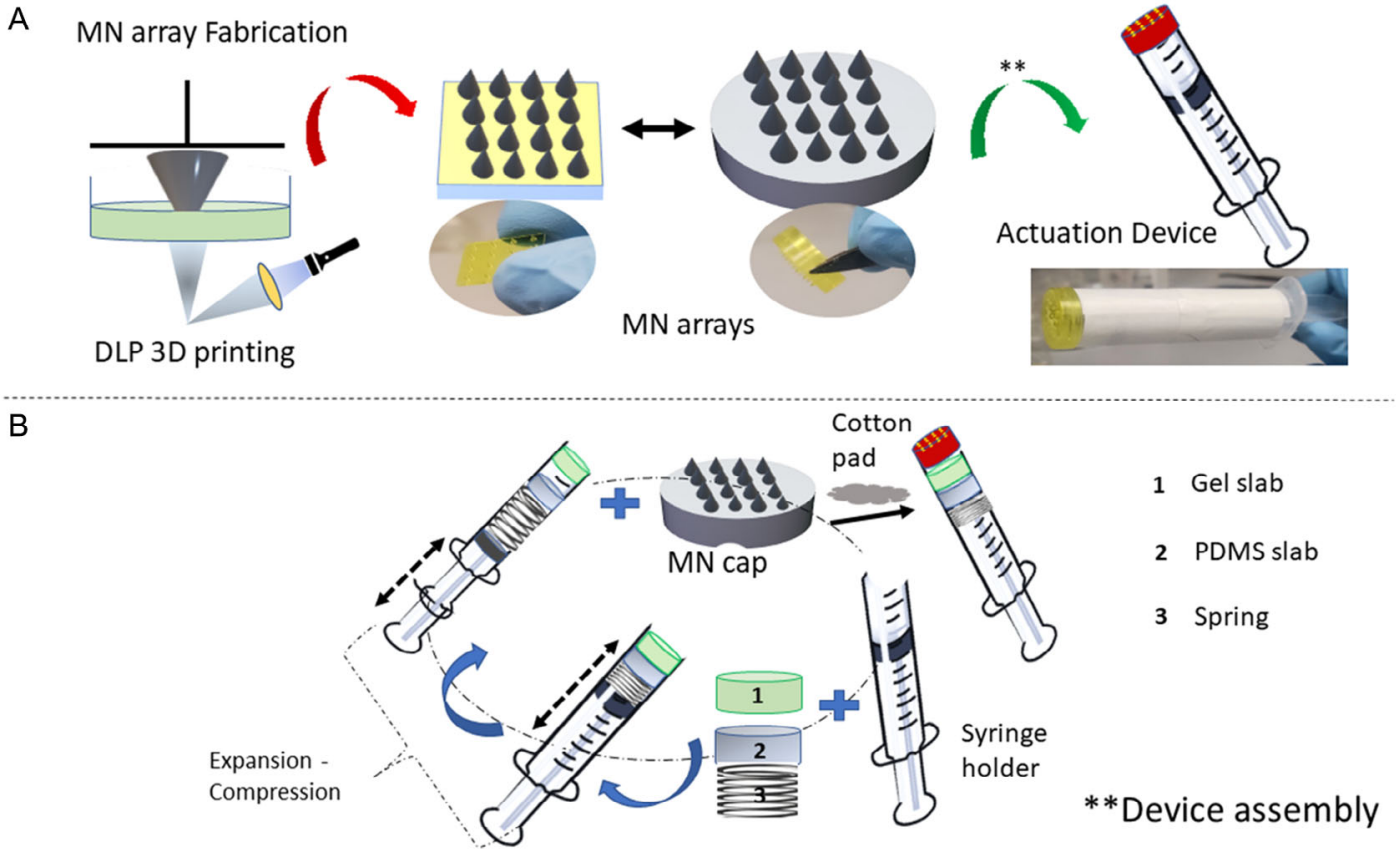
Schematic illustration of MN array preparation and microscale device assembly. A) DLP 3D printing of MN cap. B) Alignment of all of the components of microscale device. Symbols are defined as follows: 3D DLP, 3D digital light projection; MN, microneedle; and PDMS, polydimethylsiloxane.

**Figure** **1** and **2** demonstrate the development of the cost‐effective, user‐friendly, and functional biosensor design. The setup resembles a conventional medical syringe and works under the influence of a pressure gradient for body fluid extraction. The ISF collection technique described in this study relies on the synergistic action of the negative back pressure and the positive pressure applied to the skin; an oblique MN array facilitates ISF acquisition. The captured ISF is absorbed by the device through the capillary action of the thin cotton pad placed beneath the MN cap (shown in Figure 2). From there, the ISF is directed to the electrode surfaces through microchannels created in gel electrolyte. The electrodes were functionalized with GO_*x*_, LO_*x*_, and AOCE‐6 for sensing glucose, lactate, and potassium ions. The biorecognition components selectively detect the biomarkers in ISF in a concentration‐dependent manner.

#### 2.1.1 Fabrication and Characterization of MN Array

**Figure 3:**
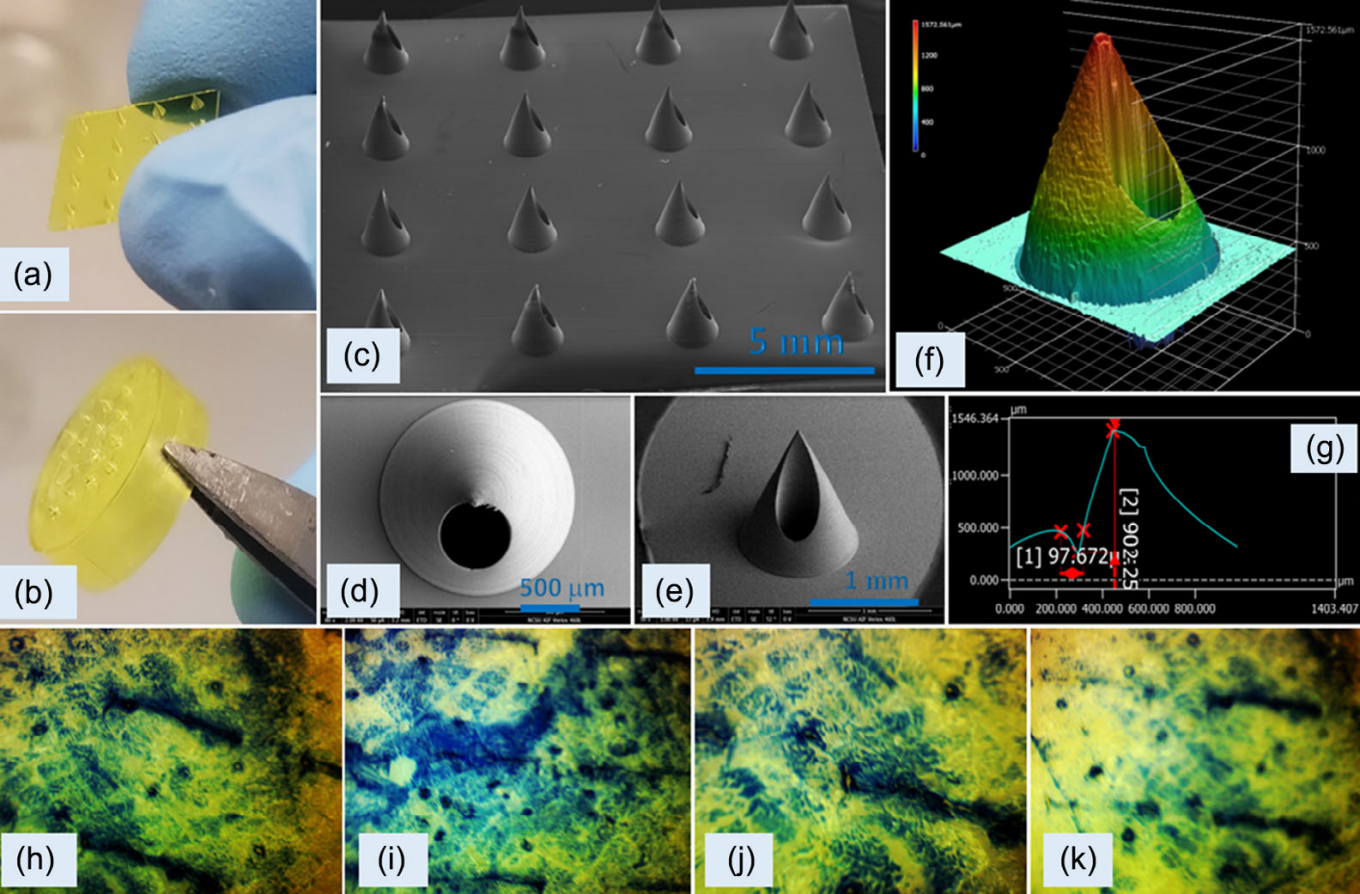
Optical images of MN arrays on a) square plate and b) cap . SEM micrographs of c) MN array, d) MN tip, and e) MN oblique view. Keyence laser scanning optical microscopy 3D image of the f) MN and g) dimensions of the MNs shown by the plot between height (*y*‐axis) and width of the needle (*x*‐axis). Optical images of trypan blue‐coated punctured porcine skin using MN arrays of height h) 750 μm, i) 800 μm, j) 900 μm, and k) 950 μm.

Figure 2 illustrates the preparation of the MN array. Each MN is conical in shape with a shank height of 900 μm; the diameter of the hollow portion of the MN is 320 mm (**Figure** **3**a,b). The cap consists of an array of 16 MNs in a 4 × 4 arrangement; the array exhibits a center‐to‐center distance of 2.5 mm between two MNs and a bore hole with a base diameter of 800 μm (Figure 3c). Scanning electron microscopy (SEM) micrographs (Figure 3c–e and S1, Supporting Information) show the characteristic topographic features of the MN array at various angles. Figure 3d,e shows SEM images of individual MN, depicting the sharpness of the MN tip and the oblique design at two magnifications. Figure 3f shows a 3D image of MN that was obtained using laser scanning optical microscopy. Figure 3g shows the detailed dimensional representation of the MNs obtained from keyence study. The needle is place upright on the stage and on exposure to laser the dimensions were recorded as such: the measurement of needle cone started at ≈500 mm including height, base diameter, tip diameter, and hollow MN diameter; these measurements are 902.5, 941.6, 9.7, and 355.2 μm, respectively. The distance between the hollow part and each side of the MN was also determined. The MN height was determined by rastering the laser in the XY plane and 0.5 nm in Z‐direction.

The DLP 3D printing process provided polymeric MNs with sharp tips.^[^ ^19^ ^]^ MNs for ISF acquisition 1) must be made of a material that exhibits a high Young's modulus value and 2) must be capable of skin penetration.^[^ ^20^ ^]^ Therefore, a yellow transparent resin containing a mixture of two methacrylate oligomers, diphenyl (2,4,6‐trimethylbenzoyl) phosphine oxide photo initiator, and lauryl methacrylate reactive diluent was used for fabricating the MN arrays. This material allows for the processing of structures with micrometer‐scale features and the straightforward removal of unpolymerized material. Shen et al. investigated the role of a cardanol‐based acrylate, UV oligomer methacrylate cardan phenolic polycondensate (MCPP), in 3D printing applications.^[^ ^20^ ^]^ Methacrylate oligomeric resins are used for 3D printing of biomedical devices, as they exhibit high Young's modulus values, high glass transition temperature (*T*_g_) values, low chemical reactivity, heat resistance, hydrophobicity, and straightforward processability.^[^ ^21^, ^22^, ^23^, ^24^ ^]^ Since these materials are hydrophobic, fewer adhesive interactions occur between the materials and biological molecules.

The hardness and Young's modulus values of the MN array material were determined using nanoindentation to be 3.29 ± 0.12 GPa (mean ± standard deviation) and 302.19 ± 10.44 MPa, respectively. These values are sufficient for piercing the porcine skin since the minimum Young's modulus value for puncturing human skin is ≈1 GPa.^[^ ^24^ ^]^ We demonstrated the skin penetration properties of the MN array with fresh porcine skin; a water‐soluble dye, trypan blue, was applied to the MN array‐treated skin for visualization of the MN array‐generated pores (as shown in Figure 3h–k). The MN arrays were inserted into the skin via manual application. The MN array penetrated the stratum corneum layer of the porcine skin without damage to the MN tips.

#### 2.1.2 Preparation and Characterization of CF WEs and Electrolyte

The device was equipped with an electrochemical sensing system consisting of two parts, CF electrodes and gel electrolyte. To satisfy the requirements of a disposable, cost‐effective, and leakproof device, solid agar gel electrolyte and CF‐based WEs (WE_1_, WE_2_, WE_3_) were used to prepare the device. The configuration of each CF electrode (WE_1_, WE_2_, WE_3_) transducer facilitated the successful detection of biomarkers. The CF WEs were stepwise functionalized for the detection of the analytes. The WEs exhibited a cross‐sectional dimension of 5 mm × 5 mm and a electrochemical active surface area (ECSA) of 0.045 mm^2^. Figure 2 shows the slabs of gel electrolyte with three sets of microchannels arranged at ≈120°.

Channels were designed to direct the analyte flow to the electrodes. The conductivity and catalytic activity of CF WEs were improved by stepwise functionalization with specific recognition molecules for glucose, lactic acid, and K^+^ detection. Li et al. suggested that coating with these types of molecules increases the electrode surface area and enhances the mass transport channels, thereby increasing the electrocatalytic ability.^[^ ^25^ ^]^ In this approach, all of the CF WEs were first smeared with a layer of siloxane ((3‐aminopropyl) trimethoxysilane (APTMS), 1 mm) and dried under vacuum to generate a stable amino‐terminated surface without compromising their electrocatalytic properties. In addition, bimetallic Pd@AuNPs^[^ ^26^, ^27^, ^28^, ^29^ ^]^ were in situ synthesized on CFs to improve their electroactivity. On an individual basis, LO_*x*_, GO_*x*_, and AOCE‐6 were immobilized via residual amino groups of APTMS on CF WE_1_, CF WE_2_, and WE_3_, respectively; CF WE_1_, CF WE_2_, and WE_3_ were used for successive multiplexed sensing of glucose, lactate, and K^+^, respectively. The amino groups of APTMS could potentially combine with the residual carboxylic group at the gamma carbon (CG)/delta carbon (CD) of the acidic amino acids like aspartic (ASP) and glutamic acids (GLU) available on the structure of enzymes (GO_*x*_/LO_*x*_) to form an amide bond. The possible reaction can proceed as shown above in Figure S2, Supporting Information, (molecules in yellow indicate ASP groups; the reaction is shown with one of them). Like the GO_*x*_ and LO_*x*_, which display binding selectivity to specific substrates, crown ethers (AOCE‐6) display binding selectivity for alkali metals (in this case K^+^).

**Figure 4:**
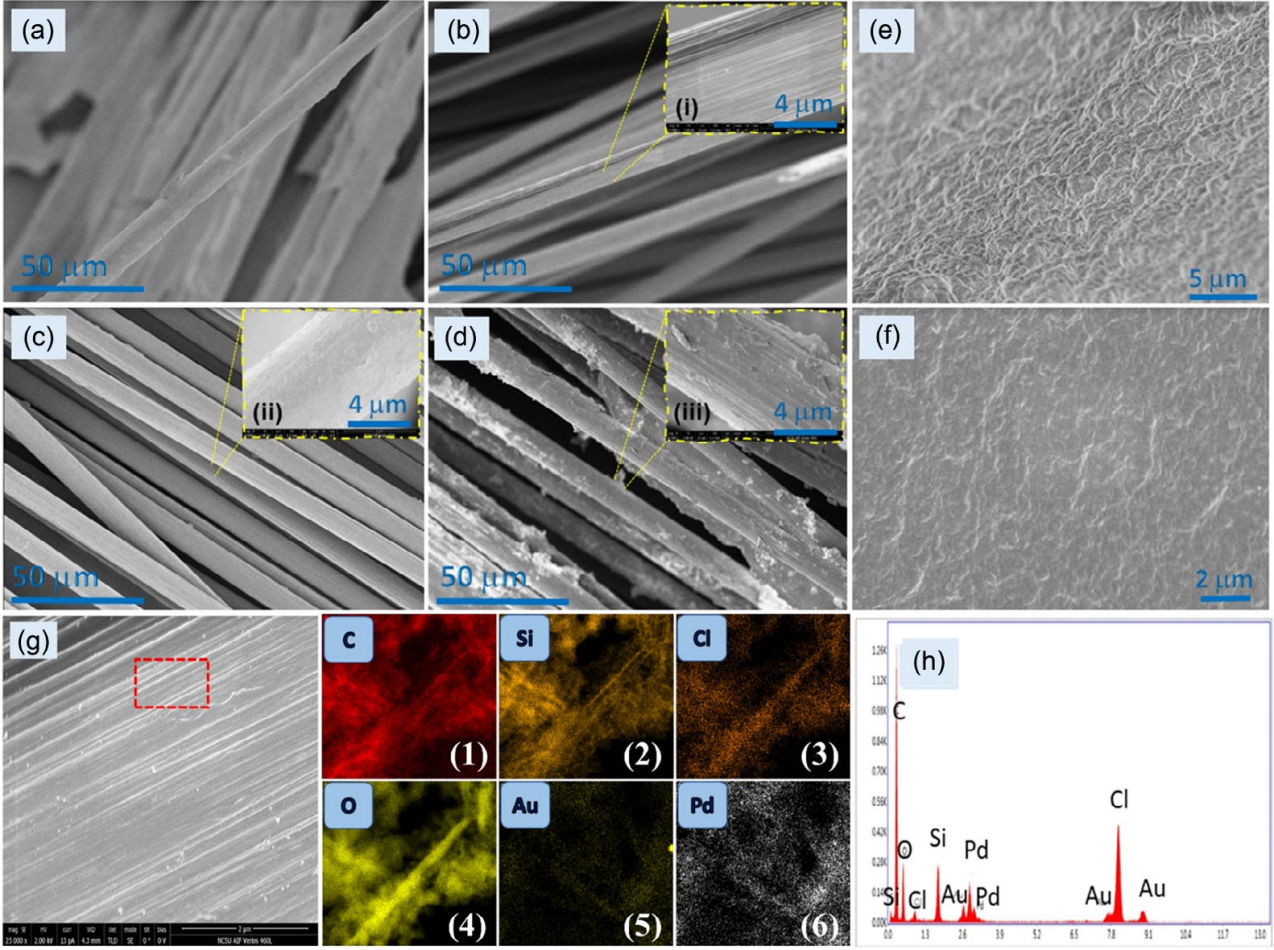
SEM images of: a) unmodified CF; b) CF/APTMS/Pd@Au/LO_*x*_, c) CF/APTMS/Pd@Au/GO_*x*_, d) CF/APTMS/Pd@Au/AOCE‐6, e–f) electrolyte gel slab, g) SEM–EDS elemental mapping of the elements 1) C, 2) Si, 3) Cl, 4) O, 5) Au, and (6) Pd, h) area EDS spectrum. Inset pictures i–iii) magnified views highlighting the coating on CF surfaces.

The AOCE‐6 cavity binds selectively with potassium ions, exhibiting ion–dipole interaction and 1:1 stoichiometry.^[^ ^30^ ^]^ The available electrophilic center of the aldehydic group of glutaraldehyde enabled the attachment of AOCE‐6 via the amino group. **Figure** **4** shows the SEM images of CF WEs at various magnifications. The topologies of the as‐spun microfibrous CF/APTMS, CF/APTMS/Pd@Au/LO_*x*_, CF/APTMS/Pd@Au/GO_*x*_, and CF/APTMS/ Pd@Au/AOCE‐6 show the in situ growth of the nanoparticles in addition to the subsequent loading of enzymes and crown ether. The CFs were observed to possess uniform size, with diameters of ≈350 nm. The low‐magnification SEM micrographs (inset pictures Figure 4(i–iii)) of CF WEs indicate nearly uniform modification, with some irregularities, including a series of grooves of various depths on the surface.^[^ ^25^ ^]^ The uncoated CF exhibited a smooth texture (Figure 4a); on the other hand, the modified CFs WEs had a rougher surface (Figure 4b) along with an uneven appearance due to the presence of particle or layer deposition. Sekar et al. proposed that these surface aberrations could widen the molecule‐to‐surface interactions and decrease the polarization of the electrode.^[^ ^31^ ^]^ Figure 4e,f shows the morphology, including the porous texture, of the agar‐based semisolid gel slab surfaces. The 3D framework of agar gel was stable and did not swell; agar is known to possess biocompatibility, antifouling properties, antibacterial properties, and electrochemical catalytic properties.^[^ ^32^ ^]^ The hydrated form of the gel encapsulated with redox species allows for electron transport through the 3D network; respective electrocatalytic conversions are triggered at lower potentials, leading to the effective performance of the device.

**Figure 5:**
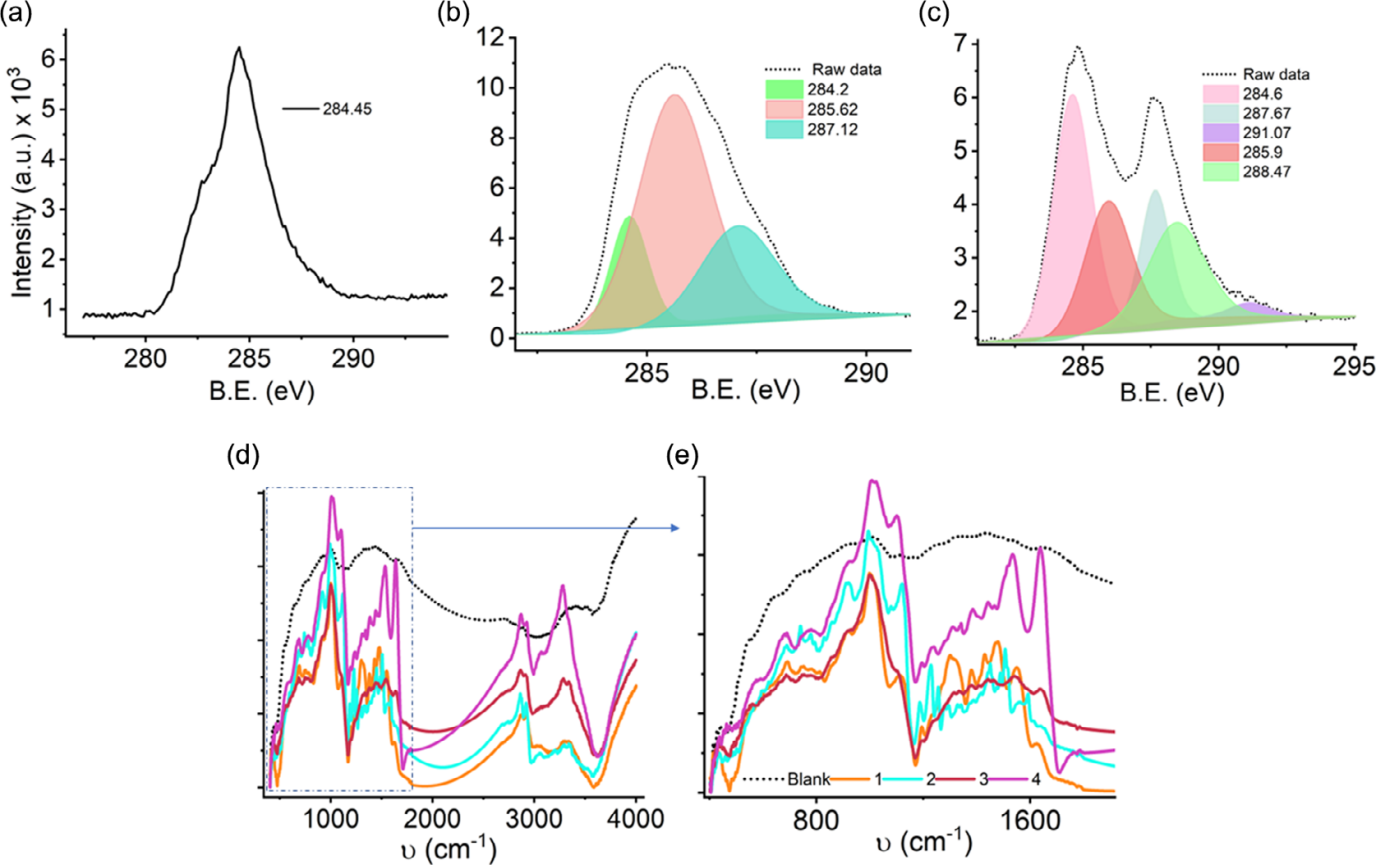
XPS C1s spectra of a) unmodified CF, b) modified CF/APTMS/Pd@Au/AOCE‐6, and c) CF/APTMS/Pd@Au/enzyme. FTIR profile of d) CF WEs and e) magnified region of Figure (d). Legends 1–4 represent CF/APTMS and CF WEs (WE_1_, WE_2_, WE_3_).

It does not solubilize the coatings on CF WEs, it can be stored at room temperature, and it is easily disposable; these attributes are appropriate for biomedical sensing. Figure 4g (1–6) and h shows the corresponding energy‐dispersive X‐ray spectroscopy (EDX) elemental mapping results. These results indicate the existence of elements C, Si, Cl, O, Au, and Pd, suggesting the dispersion of these elements on the CFs. A nearly uniform distribution of Si, O, Au, and Pd atoms could be observed throughout the examined area. Further, the modifications of CF WEs were assessed by X‐ray photoelectron spectroscopy (XPS). As shown in **Figure** **5**, modifications in the C1*s* spectra were reported after each change in the CFs.^[^ ^33^, ^34^, ^35^, ^36^, ^37^, ^38^ ^]^ The unmodified fibers show a well‐resolved sharp C 1*s* singlet at ≈285 eV (Figure 5a), which may be assigned to carbon–carbon bonded atoms in the hexagonal sheets or the graphitic structure of fibers.^[^ ^33^ ^]^ For CF/APTMS/Pd@Au/glutaraldehyde/AOCE‐6, the C1*s* spectra were fitted with peaks that were indexed at 284.2, 285.62, and 287.12 eV binding energies. These changes in standard C 1*s* spectra correspond to –C–N, –C–O, bridging interactions, which are attributed to the presence of amino‐functionalized APTMS and AOCE‐6 molecules (Figure 5b).

For APTMS/Pd@Au/LO_*x*_ or APTMS/Pd@Au/GO_*x*_‐modified CFs, the C 1*s* spectra show five signals. Four sharp peaks and one less resolved peak were noted at energies 284.6, 285.9, 287.67, 291.07, and 288.47 eV, respectively (Figure 5c). These peaks are characteristic of —C=O and β‐carbon bonding in addition to —C—N and —C—O—C bonded interactions with enzyme and siloxane molecules.^[^ ^37^ ^]^ O 1*s* spectra^[^ ^39^ ^]^ (Figure S3a,b, Supporting Information) for APTMS/Pd@Au/glutaraldehyde /AOCE‐6, APTMS/Pd@Au/LO_*x*_ or APTMS/Pd@Au/GO_*x*_ modified CF shows peaks centered at ≈533.46, 531.7, and 535.4 eV. The signal at a low binding energy (531.7 eV) belongs to Si—O bonding in the siloxane structure; peaks indexed at higher energies are related to —C=O and enzymatic interactions. A calculated increase in the C/O ratio was detected with stepwise changes to surface topology. After treatment with APTMS, the distributions of C and O were observed as 80.0 atomic wt% and 16.3 atomic wt%, respectively. For CF WE_3_, the O content increased (C/O % = 55.9/24.51); for CF WE_1_/WE_2_, the O content was also found to increase (C/O % = 51.4/46.5). On modification with siloxane‐based (APTMS) and noble metals (Pd@Au), the presence of silicon, palladium, and gold content (Figure S4a–c, Supporting Information**)** confirmed the loading of the APTMS layer and in situ growth of nanoparticles. Figure S4a, Supporting Information, shows the Au 4f spectra for the growth of monometallic AuNPs with doublet peaks 4*f*_7/2_ and 4*f*_5/2_ as indicated at 87.10 and 90.75 eV, respectively. Peak shifts of ≈3 eV in lower and 2.25 eV higher binding energy from the standard values (84.1 and 88.5 eV) were attributed to interactions with the environment. While in the bimetallic Pd@Au arrangement, the introduction of Pd led to profound lower shifts in both signals (83.37 and 86.96 eV) (Figure S3b, Supporting Information).^[^ ^38^ ^]^ Additionally, the spectrum for palladium shows the doublet with 3*d*_5/2_ and 3*d*_3/2_ peaks at 335.1 and 340.45 eV, respectively (Figure S4c, Supporting Information). Data from this study is summarized in Table S1, Supporting Information.

The Fourier‐transform infrared spectra of the CF WEs recorded are shown in Figure 5. A series of peaks was obtained for the modified CFs in the regions 3340, 3270–3290, 2850–2950, 1640–1630, 1550–1510, 1490–1440, 1380–1300, 1295–1220, 1195–1100.5, 1007, 780–750, and 695–680 cm^−1^. For enzyme‐treated (LO_*x*_/GO_*x*_) CFs, peaks indexed at 3340.95–3337.78, 2950–2850, 1636–1637.8, 1510–1549, 1450–1370, 1340–1240 cm^−1^ correspond to –O–H group stretching from proteins,^[^ ^31^, ^32^, ^39^, ^40^, ^41^, ^42^ ^]^ –C–H stretching of CH/CH_2_ groups from the graphitic backbone of CFs, >C=O groups in the enzyme, –C–N amide bands (II), –C–H bending structures, and –C–N amide bands (III), respectively. Similarly, AOCE–6‐modified CF shows peaks at 1220–1180, 2900–2860, 1500–1590 cm^−1^, which are attributed to –C–O–C stretching of the crown ether, –C–H stretching of CH/CH_2_ groups from the graphitic backbone of CFs, and –C=C stretching, respectively. Along with other CFs, APTMS‐treated blank CF shows prominent peaks for SiO_2_ and –C–Si stretching at 1020 and 1160 cm^−1^, respectively. Blank CF sonicated in the presence of mineral acid shows nonspecific broad bands in the functional region around 3020 cm^−1^, which is attributed to carbon–carbon bonds in layered CF structures.^[^ ^42^ ^]^ Shim et al. described the FTIR spectra of CFs on treatment with NaOH/organic acids, which showed the presence of carboxylic acid groups.^[^ ^42^ ^]^ The spectra with detailed peak details are shown in Figure S5A,B, Supporting Information.

#### 2.1.3 Device Assembly and Operation

The device was designed by integrating all of the components, including MN cap, thin cotton pad, micro channeled gel electrolyte, CF WEs with copper foil connectors, and hollow 1 mL syringe embedded with spring (with 1 mL outer diameter and inner diameter), through a fitted PDMS mold. Figure 2 illustrates the stepwise protocol for building and assembling the different parts of the device. All of these segments are installed within a compact 1 mL syringe‐like holder (with 1.8 cm diameter and 3.5 cm height) to create a single‐use device. Yu et al. previously prepared a miniature and disposable trinitrotoluene electrochemical sensor using polymer gel electrolyte.^[^ ^43^ ^]^ Blicharz et al. reported on the fabrication of single‐use point‐of‐care instrument for capillary blood collection, which employed a vacuum feature.^[^ ^44^ ^]^ Li et al. demonstrated a PDMS‐based hollow MN integrated with a self‐powered prevacuum actuator for a blood extraction system.^[^ ^45^ ^]^ Figure 2 demonstrates the basic arrangement and operation of the device for extraction of ISF through the pressure convection principle.

**Figure 6:**
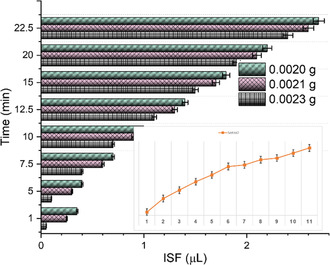
Fluid collection profile obtained from pig skin ex vivo after different durations.

One end of the spring is fixed with a solid circular PDMS mold, which is placed inside the main receptacle. The other end of the spring is attached to the second portion of the plunger‐like holder, which can mimic the function of a vacuum suction tool to withdraw fluid. After placing the electrodes at their respective locations in order to form an airtight system, all of the orifices on the MN cap and holder are sealed with superglue. For fluid retraction, the device is pressed against the porcine skin (perpendicular to MN end); the plunger prototype is pulled in the opposite direction. With the expansion of the previously compressed coil, suction (negative pressure) is created inside the device, which is backed by compaction force that is created around the MN insertion site due to the oblique geometry of MNs. The upward drag built in the system draws the ISF into MN array; the skin becomes perforated as shown in Figure 3. The liquid gets absorbed by the cotton swab through capillary action. This dual approach acts as a fluid reservoir and controls the diffusion of the sample on the electrode, thereby preventing saturation. We observed that an optimum volume of ISF was collected and free from blood traces using MNs with a height of ≈900 μm. The capped MN device typically took ≈25 min for extraction (yield ≈3.0 μL) and analysis. The fluid collection was done by the method as described in the above response. It was repeated in three batches, with same sequence of steps (summarized in **Table** **1**), and fluid collection profile over time is shown in **Figure** **6**.

**Table 1:** Fluid collection using swabs

Time [min]	Swab 1 *w*_o_ = 0.0023 g		Swab 2 *w*_o_ = 0.0020 g		Swab 3 *w*_o_ = 0.0021 g			
	*w*_*f*_	*w*_l_ */r = V*_l_	*w*_*f*_	*w*_l_ */r = V*_l_	*w*_*f*_	*w*_l_ */r = V*_l_	Average	Standard deviation
1	0.00235	5 E‐5	0.00225	2.5 E‐4	0.00245	3.5 E‐4	2.167 E‐04	1.0207 E‐03
5	0.0024	1 E‐4	0.0023	3 E‐4	0.0025	4 E‐4	2.667 E‐04	0.9837 E‐03
7.5	0.0027	4 E‐4	0.0026	6 E‐4	0.0028	7 E‐4	5.667 E‐04	0.9052 E‐03
10	0.0030	7 E‐4	0.0029	9 E‐4	0.0031	0.001	8.667 E‐04	0.8191 E‐03
12.5	0.0038	0.0011	0.0033	0.0013	0.0035	0.0014	1.267 E‐03	0.7127 E‐03
15	0.0042	0.0015	0.0037	0.0017	0.0039	0.0018	1.667 E‐03	0.6075 E‐03
20	0.0047	0.0019	0.0041	0.0021	0.0043	0.0022	2.067 E‐03	0.5 E‐03
22.5	0.0052	0.0024	0.0046	0.0026	0.0048	0.0027	2.567 E‐03	0.3535 E‐03

### 2.2 Electrochemical Characterization of CF WEs

**Figure 7:**
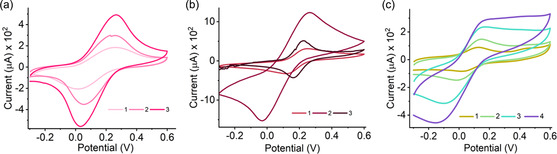
Electrochemical characterization of CF WEs before and after stepwise modification. CV plots for a) CF/APTMS/Pd@Au/LO_*x*_, b) CF/APTMS/Pd@Au/GO_*x*_, and c) CF/APTMS/ Pd@Au/AOCE‐6 electrodes recorded in semisolid electrolyte gel slab. Legends 1–3 represent the voltammograms after each functionalization. Legend 4 in Figure (c) was obtained after adding KCl solution.

CF WEs were characterized by the CV technique at all stages of functional modification in a 15 × 15 × 5 mm gel electrolyte block encapsulated with the redox couple [Fe (CN)_6_]^3−/4−^. **Figure** **7** shows a representation of the CV responses on discrete electrodes. The voltammetric signals on inactivated blank CF electrodes showed poorly defined cathodic and anodic peaks, suggesting electrochemical irreversibility. In comparison, the reversible behavior appeared to improve after chemical modifications. The symbolic oxidation and reduction signals at ≈0.15 and −0.1 V were hierarchically resolved due to the increase in the electroactive surface area (0.0115–0.315 cm^2^).^[^ ^16^ ^]^ The corresponding ECSA values were calculated at every stage in terms of double‐layer capacitance values using the following relation.

(1) ECSA = Cdl/Cs

The electroactivity of the CF WEs and the gradual improvement in redox electrochemistry of homogeneous Fe [(CN)_6_]^3−/4−^ were demonstrated in the following order: CF–Pd@Au–GO_*x*_/LO_*x*_/AOCE‐6 > CF–Pd@Au > blank CF. For CFs WE_1_, WE_2_, and WE_3_, the loading of metal catalyst led to an improvement in the signal symmetry of the redox pair, with an amplification in anodic/cathodic peak current (*I*_pa_/*I*_pc_) of ≈58.9/67.8 μA and voltage separation of 0.248 V (Figure 2b and S6, Supporting Information). The resulting enhancement in current responses and shifting to lower potential validate the loading of self‐assembled layers of the active materials.

Further, the presence of the enzyme showed a significant effect on the current responses for CFs (WE_1_, WE_2_) due to rapid electron transfer from the enzyme active centers.^[^ ^46^ ^]^ An approximate twofold increase in current density was obtained for both GO_*x*_‐ and LO_*x*_‐modified electrodes (≈160.2/166.3 and 210.5/270.9 μA, respectively); peak separations of 0.103 and 0.201 V, respectively, were noted. AOCE‐6‐coated CF (WE_3_) exhibited substantial enhancements in current responses such as 144.5/233.9 μA, respectively, with a potential gap of 0.031 V. Lower onset potentials and high Ipa/Ipc ratios resulted from superior charge transfer kinetics and rapid electrochemical reversibility. The faster kinetics on modified electrodes confirmed the ability to detect the analytes under smaller potential windows. The redox signal was improved with each increase in K^+^ concentration, as shown by the optimization curve in Figure S6, Supporting Information; gradual peak shifts toward lower potentials were noted. This result is attributed to the action of the positive electrode surface, which encouraged the oxidation/reduction of receptor ion [Fe(CN)_6_]^3−/4−^ and facilitated the shuttling of electrons across the immobilized catalyst layer. This finding concurs with the finding previously reported by Kumbhat and Singh on electron transfer kinetics across the 11‐MUA monolayer for potassium ion sensing.^[^ ^47^ ^]^ The improvement in the reversibility of redox voltammetry may be attributed to regulation in proximal diffusion layers across CF WEs. Wightman proposed that if the area of the receptive region on WEs falls intermediate between that of a micro‐ and macroelectrode, the pattern of diffusion can be either linear or radial.^[^ ^48^ ^]^ Janisch et al. explained the correlation of diffusion type, electrode size, and sweep speed of the signal.^[^ ^49^ ^]^ At a constant scan rate (2 mV s^−1^), the peak reversibility improved with the successful stepwise coating of the electrodes. This result shows the transformation of initial hemispherical diffusion to the linear configuration. Thus, the thickness of diffusion layers decreased, which allowed better mass transport. Additionally, gradual switching to the rectilinear form of diffusion from radial (overlapping distribution) could potentially reduce the cross‐talk between the electrode system adopted here.^[^ ^9^ ^]^ Moreover, the precedent model of transport properties was further studied computationally via time‐dependent COMSOL studies.^[^ ^50^, ^51^ ^]^

### 2.3 Amperometric Sensing

**Figure 8:**
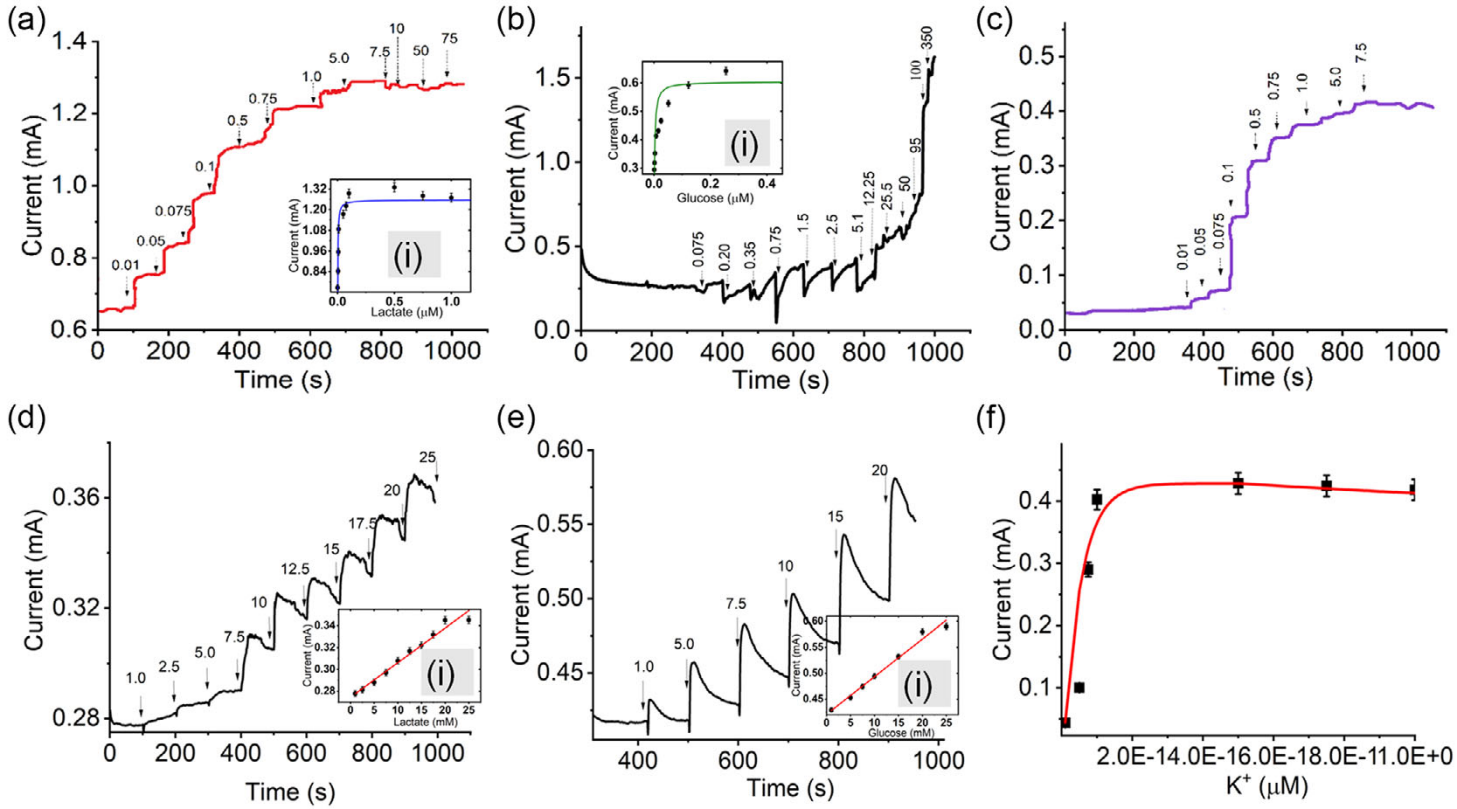
Amperometric responses of a,d) APTMS/Pd@Au/LO_*x*_, b,e) APTMS/Pd@Au/GO_*x*_, and c) APTMS/Pd@Au/AOCE‐6‐coated CFs. Inset pictures show the corresponding calibration curves of current responses versus concentration of analytes, i) enzyme kinetics for LO_*x*_/GO_*x*_ by the Michaelis–Menten model. f) is the exponential decay curve model for potassium ion detection.

To optimize the detection of lactate, glucose, and K^+^ ion levels with LO_*x*_, GO_*x*_, and AOCE‐6‐modified CF WEs (WE_1_, WE_2_, and WE_3_), a current versus time (*I–T*) electroanalytical technique called amperometry was employed under optimum conditions. **Figure** **8**a–e shows the amperometric *I–T* responses for APTMS/Pd@Au/LO_*x*_, APTMS/ Pd@Au/GO_*x*_, and APTMS/Pd@Au/AOCE‐6, deposited CFs.

Three *I–T* curves were recorded for in vitro detection of lactate, glucose, and K^+^ with working potentials fixed at 0.3, 0.2, and 0.25 V, respectively. A wide range of concentrations were evaluated: 100 μm–75 mm of lactic acid, 0.05–350 μm of D‐glucose, and 0.01–10 μm of KCl solutions in PBS. 5 μL of analytes were added at regular intervals of ≈75 s after an incubation time of ≈150 s (WE_1_), 300 s (WE_2_), and 350 s (WE_3_). A steady amperometric *I–T* curve with a well‐defined amplification in current was observed on the addition of 0.01 (a), 0.05 (b), 0.075 (c), 0.1 (d), 0.5 (e), 0.75 (f), 1.0 (g), and 5.0 μm (h) of lactate as shown in Figure 8a. There were immediate enhancements in the current volume at every dose of analyte injection. For WE_2_, the amperometric *I–T* curve showed a slow increase in current volume initially on the addition of 0.075 (a), 0.20 (b), 0.35 (c), 0.75 (d), 1.5 (e), 2.5 (f), 5.1 (g), 12.25 (h), and 25.5 μm (i) of glucose; steep increments were recorded on supplying 50 (j), 95 (k), 100 (l), and 350 μm (m), as shown in Figure 8b. The peak value near the step increased since the concentration of the solution did not reach the equilibrium state instantaneously immediately after adding glucose.^[^ ^24^ ^]^ For potassium ions, the amperometric signal appeared to strengthen slowly over time (Figure 8c) on the introduction of 0.01 (a), 0.05 (b), 0.075 (c), 0.1 (d), 0.5 (e), 0.75 (f), 1.0 (g), 5.0 (h), and 7.5 μm (i) of KCl. The corresponding calibration plots for WE_1_ and WE_2_ [Figure 8a(i),b(i)] were fit to the enzyme kinetics function using the Michaelis–Menten model. This plot determines the conversion rate of lactate or glucose to pyruvate or gluconate, respectively, and H_2_O_2_ by enzyme catalysis. Based on this model, the current response to substrate concentration was calculated using the equation

(2) $$y_{i}=V_{\text{max}}\left[x\right]/K_{m}+\left[x\right]$$

In this equation, *y*_*i*_ is the reaction rate or current response in this study, [*x*] is the substrate (lactate/glucose) concentration, *V*_max_ is the maximum rate obtained, and *K*_m_ is Michaelis–Menten constant or substrate affinity of the enzyme.^[^ ^9^ ^]^ The derivative curves obtained for both WE_1_ and WE_2_ show a steep increase in the rate of reaction with increasing substrate concentration. This result is attributed to the faster turnover rate and unsaturation of the catalytic site of the enzyme. The corresponding values of *V*_max_ and constant *K*_m_ were calculated as 1.2562 ± 0.03 and 1.223 × 10^−5^ for WE_1_ and 0.6043 ± 0.07 and 1.708 × 10^−7^ for WE_2_, respectively. For K^+^ detection, the respective nonlinear plot (Figure 8f) was fitted to an asymptomatic nonlinear regression model.

The estimated lower values of *K*_m_ for enzyme kinetics were attributed to faster conversion of substrate and the availability of exposed active sites. The steep response of reported biosensors (WE_1_, WE_2_, and WE_3_) was characterized with high sensitivities of 1.258, 0.549 and 0.657 μA μm ^−1^ cm^−2^ and low detection limits of 0.01, 0.080, 0.05 μm, respectively (with a probability value more than the *F* value from statistical analysis). Previous studies on glucose and lactate monitoring involved devices with lower sensitivity. Additionally, the interference studies were performed using 100 μm of uric acid, ascorbic acid, dopamine, and glucose (Figure S7, Supporting Information). A well‐defined proportionate increase in current was recorded for the concentration range of 0.005–0.75 μm of lactate. The results show that the presence of other analytes (e.g., uric acid, ascorbic acid, dopamine, and glucose) did not show much drift in current response; the enhancement in the current signal was observed on adding 1.0–5.0 μm of lactate. The amperometric signals were recorded for both WE1 & WE2 were also recorded in the range 1–25 mm of lactate (Figure 8d,e(ii)) and glucose (Figure 8e,e(i)). Detection limits of 0.73 and 0.52 mm were obtained for WE1 and WE2

Recently, Chen et al. reported a sensitivity of 18.0 μA cm^−2^ mm ^−1^ over the working range of 0–100 μm for sensing glucose in sweat.^[^ ^52^ ^]^ Wang et al. estimated the following essential parameters for the MN‐based sensor: *K*_m_ = 8.18 (±2.30) mm and *V*_max_ = 6.55 (±1.17) μA.^[^ ^9^ ^]^ Lee et al. compared the activity of enzymatic and nonenzymatic glucose sensing using an Au–Ni alloy electrode. The sensitivity and LOD of the enzymatic and nonenzymatic sensors were observed as 1.30 μA mm ^−1^ and 0.29 μm for the enzymatic sensor as well as 0.96 μA mm ^−1^ and 5.84 μm for the nonenzymatic sensor, respectively.^[^ ^53^ ^]^ Kim et al. described non‐enzymatic sensing of lactate using a NiO and Ni(OH)_2_‐based electroactive surface, which showed sensitivities of 9.08 and 35.76 μA (mm cm^2^)^−1^ and detection limits of 0.53 and 0.59 mm, respectively^[^ ^54^ ^]^; these values are severalfold lower than those described the present study. A detailed comparison of sensor performance is provided in **Table** **2**. Also, for immobilized systems like these, the apparent *K*_m_ is calculated in terms of diffusion of substrate. As such, the Michaelis–Menten kinetics is the one of the simplest and best‐known models for the nonlinear reaction diffusion process (details are provided in the Experimental Section). The value of apparent *K*_m_ was calculated as 0.25 and 1.5 mm for LO_*x*_ and GO_*x*_, respectively, using the *V*_max_ 1.256 mAcm^−2^ (LO_*x*_) and 0.6043 mAcm^−2^ (GO_*x*_). In the crosstalk test, in a one‐by‐one manner, all of the three WEs (WE_1_, WE_2_, WE_3_) and the separate counter and reference electrodes were connected to potentiostat input. Only one WE was used at a given time. Since the WEs were connected to the virtual ground when they were not in use, no interelectrode crosstalk was observed. It was demonstrated that disconnecting each WE sequentially in this test did not cause any intermittent noise in the readings of the surrounding electrodes (Figure S8, Supporting Information) since the WEs were connected to the virtual ground when they were not in use.

**Table 2:** Comparison of recently reported lactate and glucose biosensors with the proposed device

Lactate Biosensor					
WE	Medium	Sensitivity	LOD	Conc range	Ref
Au/LOD	PBS/blood plasma	2.15 μAmm ^−1^/0.337 μAmm ^−1^	1.54 mm	1–30 mm/5–30 mm	[9]
Cysteamine/AuNP	PBS/FBS	31.40 μAmm ^−1 ^cm^2^/73.16 μAmm ^−1^ cm^2^	411 μm	500 μm − 7 mm/500 μm − 5 mm	[46]
Ni/TiO_2_/Gr	PBS		19 μm	50μm − 10 mm	[55]
SPCE/GO/NAD+/Fe (CN)_6_^3−^	PBS	0.14 μAmm ^−1^	–	0.25–4 mm	[56]
Au/MWCNTs/MB	Artificial ISF	1473 μAcm^−2^ mm ^−1^	2.4 μm	10–200 μm	[57]
polycarbonate/LOD	human sweat	–	0.2 mm	0–7 0 mm	[52]
PtNp‐CNF‐PDDA/SPCEs/LOD	human sweat	36.8 mA m ^−1^ cm^2^	11 μm	25–1500 μm	[53]
Wearable device/MN	ISF	–	0.15 mm	0–100 mm	[58]
CF WE_1_	in vitro/ISF	1.258 mA μm ^−1^ cm^−2^	0.01 μm	100 μm–75 mm	this work
Glucose Biosensor					
Wearable device/MN	ISF	–	0.32	0–28 mm	[58]
GP‐hybrid device	human sweat	1 μAmm	0.01 mm	0.01–0.7	[59]
E‐ring/carbon electrode	human sweat	23 nA μm	1.2 μm	12.5–400 μm L^−1^	[80]
Self‐powered watch	human sweat	3.29 nA μm	–	0.05–0.2	[81]
Cu‐RGO paper electrode	PBS	0.050399 μA μm ^−1^	0.5 μm	2–2000 μm	[82]
Pd–Pt core–shell NCs	PBS	0.170 μA μm ^−1 ^cm^−2^	41.1 μm	300–6800 μm	[83]
Ag‐0.5%@ZIF‐67/GCE	PBS	0.379 μA μm ^−1^ cm^−2^	0.66 μm	2–1000 μm	[60]
MoS_2_/Au NPs/GO_*x*_	PBS	13.80 μA μm ^−1^ cm^−2^	0.042 mm	0.25–13.2 mm	[61]
CF WE_2_	in vitro/ISF	0.549 and 0.657 μA μm ^−1^ cm^−2^	0.080 μm	0.05–350 μm	this work

### 2.4 Ex vivo Performance of the Biosensor

**Figure 9:**
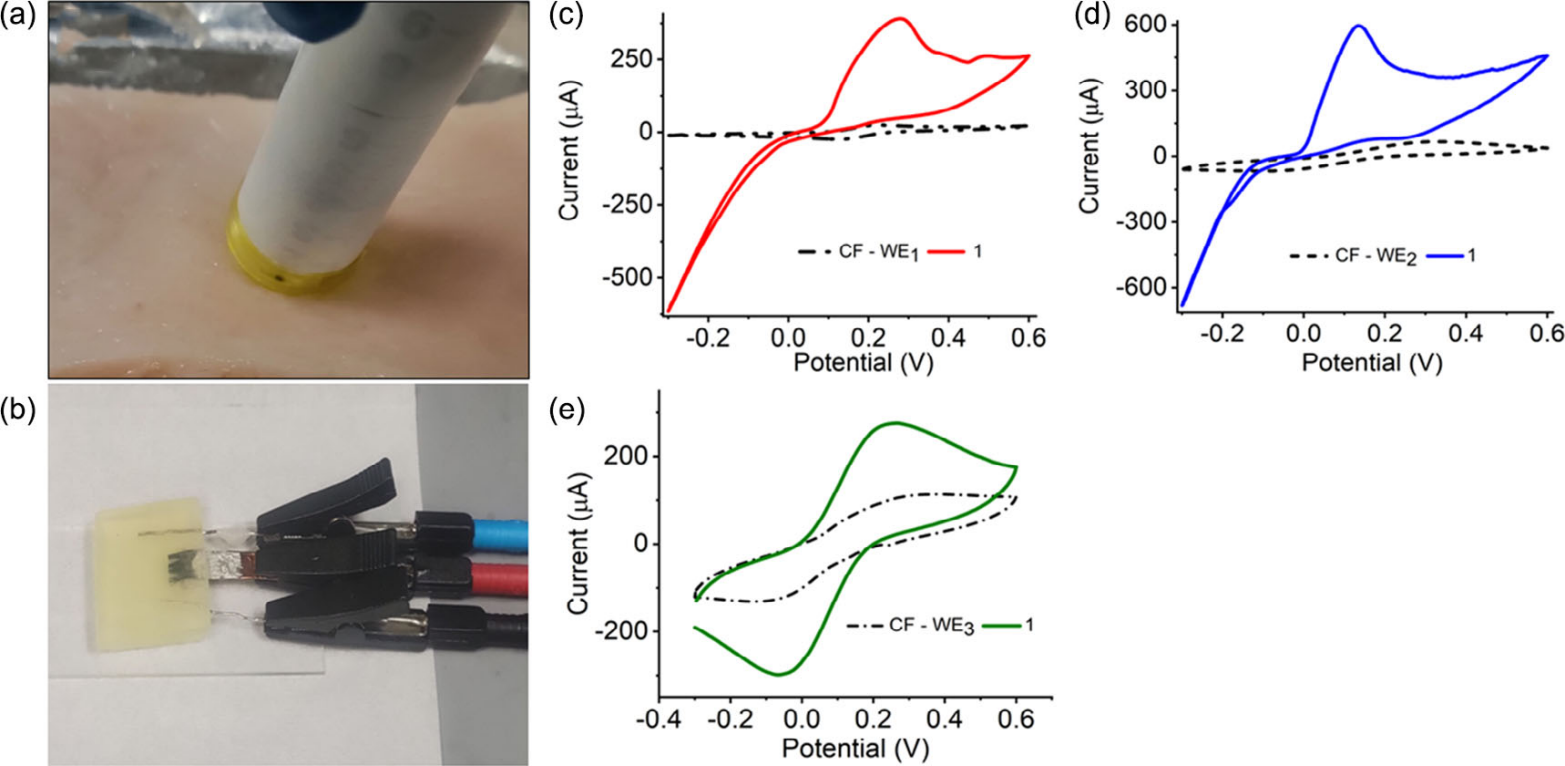
Ex vivo testing of the device on porcine skin. Optical images of a) applying the device on skin for insertion and collection of ISF, and b) three‐electrode system for transdermal monitoring of analytes. CV plots for measuring ISF: a) glucose, b) lactate, c) potassium ion levels. Legend 1 in Figure (c–e) represents the introduction of analytes.

The proof‐of‐concept CF‐based three‐in‐one biosensor was tested with an ex vivo porcine skin sample. Fresh porcine skin was used for obtaining real‐time biomarker measurements as the content of porcine ISF is analogous to that of ISF in humans.^[^ ^5^, ^11^, ^13^ ^]^ The porcine skin was shaved and cleaned before the application of the MN‐based device for ISF extraction. **Figure** **9**a shows the insertion of the device into the stretched skin. The collected fluid was analyzed by connecting the respective electrodes (WE, CE, and RE). The CV responses from *ex vivo* studies were compared with those of in vitro samples to understand the concentrations of the biomarkers. Comparative cyclic voltammograms of the CF–Pd @AuNPs/GO_*x*_/LO_*x*_/AOCE‐6 (WE_1_, WE_2_, and WE_3_) electrodes in the absence and presence of glucose, lactate, and K^+^ are shown in Figure 9; a dotted curve was used to indicate the modified CF electrodes. The CV curves of both WE_1_ and WE_2_ showed forward oxidative waves, corresponding to the irreversible oxidation of lactate and glucose.^[^ ^52^, ^53^, ^55^, ^56^, ^57^, ^58^, ^59^ ^]^

For WE_1_, an increase in the anodic peak current was observed at ≈0.31 V, indicating the onset of lactate oxidation to pyruvate and H_2_O_2_. (Figure 9c). For WE_2_, an amplification in oxidation current was observed at ≈0.18 V, marking the onset of glucose oxidation to gluconic acid and H_2_O_2_. These current signals continued to increase further up to an applied potential of 0.60 V. Additionally, there was corresponding increase in cathodic current in the reverse sweep of the CV curve for both WE_1_ and WE_2_, which is attributed to the parallel reduction of generated H_2_O_2_ in the system at the onset potentials of 0.08 and 0.01 V. Based on this finding, the device allows the oxidation of biomolecules at lower potentials than those reported in previous studies.^[^ ^60^, ^61^, ^62^, ^63^ ^]^ As shown in Figure 8, the peak current for WE_1_ was lower (≈370 μA) than the increase in current response (≈610.29 μA) for WE_2_ due to the low lactate level in ISF.

The low current density for the LO_*x*_‐modified electrode is attributed to the low concentration of lactate, which correlates with the 0.01 mm lactate signal; the glucose content is similar to a fasting blood sugar level (3.15 mm). The performance of the sensor for detecting K^+^ levels was also evaluated. The relative responses of augmenting and decaying rates as a function of glucose uptake, glycolysis, and glycogenesis were observed (Figure 9). A high carbohydrate diet needs to be supplemented with potassium enrichment to facilitate glycogenesis and glucose homeostasis. A stimulated current signal of +290.789/−318.71 μA was recorded on WE_3_, which corresponded to the K^+^ level. The volume of current obtained for K^+^ coincides with 6.5 mm (7.5 mEq L^−1^) in vitro, which is higher than the physiological concentration (3–5 mEq L^−1^ in ISF). This finding coincides with a glucose level of 7.1 mm of its PBS solution, which in turn is considerably higher than standard ISF concentration (3.9–5.6 mm). It is anticipated that this device can be used to better understand the performance of the sensor with food consumption, exercise, and fasting.^[^ ^58^ ^]^ Also, the effect of height (300–900 μm), of these types of needles on the bleeding from the animal or human skin, has not been addressed in this study and will possibly be investigated from in future in vivo studies.

### 2.5 Numerical Modeling for Analyte Transport

**Figure 10:**
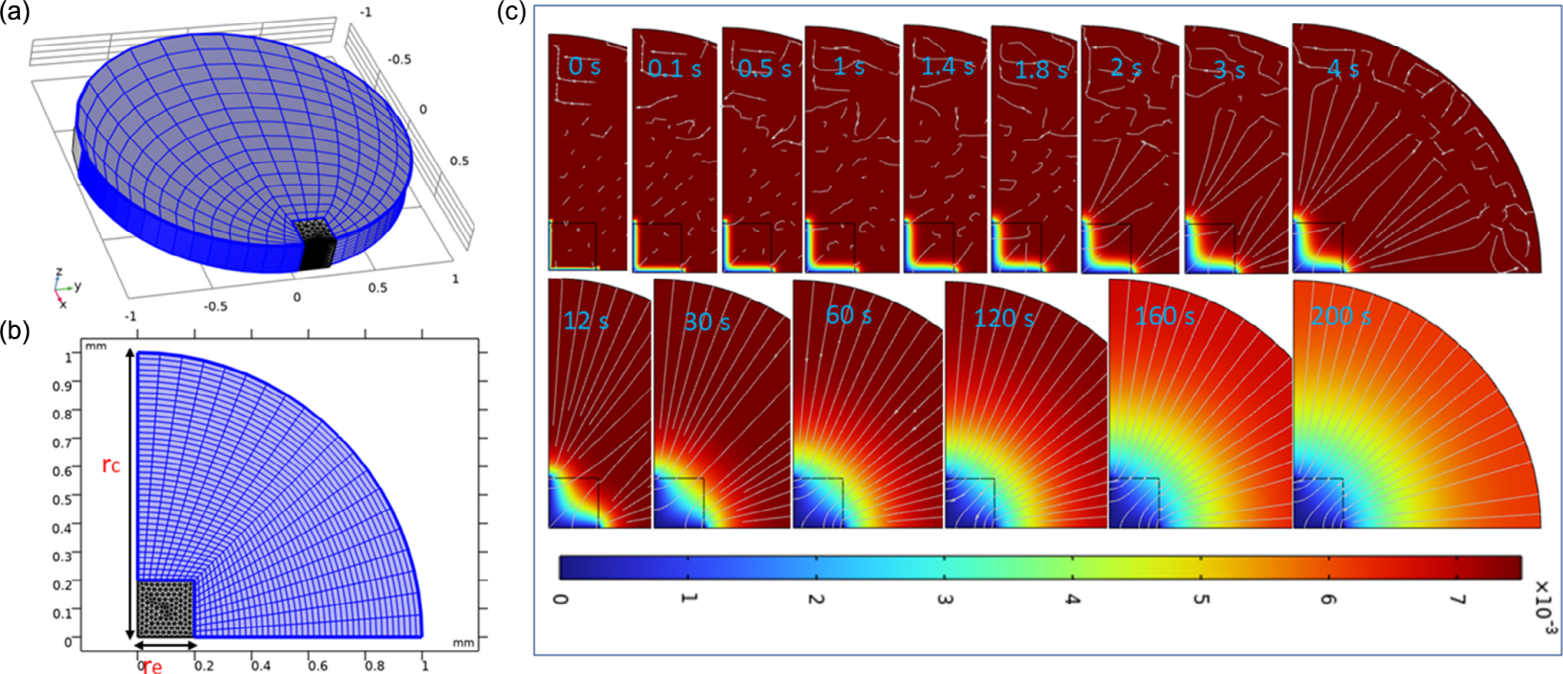
a) Schematic design of the simulated electroactive space in electrolyte, b) 2D domain of the microsensor electrode radius (*r*_e_), and c) color maps showing the expansion of the diffusion layer over time (0–200 s).

**Figure** **10**a,b shows the 3D and corresponding 2D symmetric work model or confined space representing the cross section of the working electrochemical cell in COMSOL Multiphysics (COMSOL Inc., Stockholm, Sweden). The proposed device operates in several phases; in particular, analyte diffusion on the electrode surfaces is crucial for the sensing process.^[^ ^17^ ^]^ Therefore, surface diffusivity inside the gel slab was estimated in a time‐dependent manner. In this simulation, ISF was diffused from the transdermal area to the inside of the electrolyte slab through capillary action of the cotton pad and then through a single curved channel to the electrode surfaces.^[^ ^62^ ^]^ Research groups, including Tamba et al. and Karal et al., previously performed experimental and simulation studies to analyze the diffusion of drugs through channels.^[^ ^63^, ^64^ ^]^

It is important to estimate the transport behavior in a sensing device in a similar context. Figure 10c shows the typical simulation results of the transportation of fluid via flux in the defined space. Before onsetting the molecular transport computation, the contrast of the cell was the same throughout the allotted boundaries, as shown in Figure 10c at 0 s. The movements of diffusion currents were reflected in terms of the contours with undefined margins using a color pattern (0–7), as evident from Figure 10c. With an increase in time from 0.1 to 200 s, the currents from the red color region moved faster toward the demarcated electrode region and the color gradient moved in the opposite direction, showing complete coverage of the electroactive surface by analyte with time. Corresponding experimental evidence from amperometry curves in Figure 8 shows the increase in current with the available concentration of the analytes.

Further, the flux of a substance across a front, J (mol m^−2^ s), is given by Fick's law relation

(3) $$\frac{\partial c_{i}}{\partial t}=D_{i}\;\nabla^{2}c_{i}-v\nabla c_{i}$$

where *P* (m s^−1^) is the permeability coefficient of fluorescent probes in the nanopore, *C*_*i*_ is the concentration of the analyte entering in a time ‘*t*’ dependent manner, and *D*_*i*_(m^2^ s^−1^) is the diffusion coefficient of the analyte in the cell. Here, we selected a concentration gradient across the cell from outside (MN array–cotton pad) to inside (electrode surface in gel slab). A similar approach was reported by Sharmin et al. for understanding the diffusion of oligoarginines along with a fluorescent probe through a permeable membrane to study the drug delivery rate.^[^ ^65^ ^]^ The time of diffusion was studied in terms of Einstein's approximation, which is given as

(4) $$t\approx x^{2}/2D_{i}$$

where *x* is the distance covered by the analyte in a nonlinear manner (in this study) following Nernst–Planck–Poisson equations after passing time *t* via microchannels and capillary action. Later, the analyte is anticipated to be distributed homogenously in the simulated 2D space or corresponding experimental 3D gel slab.

## 3 Conclusion

In summary, we report on the fabrication of an integrated device for minimally invasive monitoring of lactate, glucose, and K^+^ ion levels. Our device includes MN array‐supported ISF withdrawal via transient convective force and selective biosensing on flexible CF electrodes that were modified with biorecognition factors. Each component of this first of its kind model device is coordinated to enhance the operation of the device, which provides an option for painless transdermal diagnosis. The MN array cap prevents a movement of air inward once the outlets along the device body are sealed. A low‐pressure region developed inside the device, which facilitated skin puncture and ISF flow. The straightforward design and workflow of our device does not require skilled labor. Based on the data presented in this study, we intend to carry out experiments on human participants under different daily activities to assess the performance of the device.

## 4 Experimental Section

4.1

4.1.1

##### Materials

PAN‐based CF was obtained from E&L Enterprises (Oakdale, TN, USA). 3‐aminopropyl trimethyl siloxane (APTMS), 2‐ (3, 4‐epoxycyclohexyl) ethyltrimethoxy silane (EETMS), palladium acetylacetonate (II) [Pd(acac)_2_], tetrachloroauric (III) acid trihydrate, formaldehyde solution (30%), glucose oxidase (GO_*x*_, from *Aspergillus* *niger*, 10 KU), D‐(+)‐ glucose anhydrous, lactate oxidase (LO_*x*_, from *Aerococcus viridans*, 10 − 40 U mg^−1^), bovine blood, L‐lactic acid, microbiology grade agar agar powder, poly(ethylene glycol), hydrochloric acid (HCl), polyvinyl chloride (PVC), phosphate buffer solution (PBS) (1.0 m, pH 7.4), potassium chloride (KCl), sodium dihydrogen phosphate (NaH_2_PO_4_), potassium ferricyanide, silver wire (diameter 0.5 mm, 99.9% trace metals basis), 4‐aminobenzo‐18‐Crown‐6 (AOCE‐6, 98%), diphenyl (2,4,6‐trimethylbenzoyl) phosphine oxide photo initiator, lauryl methacrylate, and syringe assembly were purchased from Sigma‐Aldrich (St. Louis, MO, USA). Polydimethylsiloxane (PDMS) elastomer base and curing agent were obtained from Dow Corning (Midland, MI, USA). A copper foil single‐sided electrically conductive tape (0.065 mm × 50 mm × 25 m), electrically conductive epoxy silver adhesive, and room‐temperature cure (Air Dry AA‐Duct 907, 5 g) were obtained from Amazon (Seattle, WA, USA). Pig skin was obtained from a commercial abattoir (City Packing Company, Burlington, NC, USA) after receipt of a research specimen permit from the North Carolina Department of Agriculture and Consumer Services (dated December 8th, 2021).

##### Designing MN Array

All MN arrays (plate‐ and cap‐like structures) were designed using Solidworks 2016 software (Dassault Systèmes, Vélizy‐Villacoublay, France) and fabricated using commercial S130 3D printer (Boston Micro Fabrication, Maynard, MA, USA), which utilized digital light projection technology. A biocompatible resin named photoreactive resin BIO made by Boston Micro Fabrication was used for MN fabrication. This yellow transparent resin is a mixture of two methacrylate oligomers, diphenyl (2,4,6‐trimethylbenzoyl) phosphine oxide photoinitiator and lauryl methacrylate reactive diluent. This resin was assessed by the manufacturer using the ISO 10 993‐10: 2010, ISO 10 993‐12: 2012, and ISO 10 993‐2: 2006 skin irritation test; the ISO 10 993‐12: 2012 in vitro cytotoxicity test; as well as the ISO 10 993‐12: 2012 acute systemic toxicity test. It was postcured with a 405 nm wavelength lamp (Formlabs Inc., Somerville, MA) for 10 min at 45°C. The height of the MN was 900 μm, and the base diameter of the MN was 800 μm. The diameter of the hollow part of the MN was 320 μm. The base of MN thickness was 130 μm.

The dimensions (e.g., height, base diameter, tip diameter, and hollow diameter) of the MN were assessed using a VK‐X250 3D laser scanning confocal microscope (Keyence, Tokyo, Japan); laser confocal optics were used for field depth measurement. A 408 nm wavelength laser source was used for imaging. There were 16 hollow MN in each 4 × 4 MN patch, which exhibited a MN interval distance of 2.5 mm. MN patches were disinfected with sterile 70% isopropyl alcohol (VWR, Radnor, PA, USA). To obtain hardness and Young's modulus values from the MN material, nanoindentation was performed. A Ubi‐1 Nanoindenter (Hysitron, Minneapolis, MN, USA) with Berkovich‐type tip was used for the nanoindentation measurement; a maximum force of 1000 μN, a loading time of 20 s, a dwell time of 10 s at maximum load, and an unloading time of 20 s were used in the study. These steps were repeated for 10 locations on the material. Through Oliver–Pharr analysis of the unloading curves, the hardness and Young's modulus values were obtained.^[^ ^3^, ^66^ ^]^

##### Fabrication of Carbon Fiber Working Electrodes

Prior to modification, the pristine CFs were activated as follows. The CFs (0.05 g) were immersed in HCl (1 N) solution and sonicated for 20 min. The CFs were rinsed with double‐distilled Millipore (DDM) water, immersed in isopropyl alcohol (3 mL), and cleaned by sonication for 30 min. The fibers were again washed with DDM water and dried in a vacuum oven at 60 °C for an hour. The modification of the CFs to give functionalized WEs (APTMS/CF; APTMS/CF/GO_*x*_; APTMS/CF/Pd@Au/GO_*x*_; APTMS/ CF/LO_*x*_; APTMS/CF/LO_*x*_/Pd@Au; APTMS/CF/AOCE – 6; APTMS/ CF/AOCE – 6/Pd@Au) was performed as follows. The CF bundle was treated with ethanolic solution of APTMS (1.5 m), by dipping for 20–30 min at room temperature. The bundle was flattened on a glass slide using tweezers and dried at 60 °C. The CFs obtained after treatment at an elevated temperature appeared as a flat, hard, and malleable material.

For APTMS/CF/GO_*x*_ or APTMS/CF/LO_*x*_, the fabricated APTMS/CF was further treated with 3 μL solution of GO_*x*_/LO_*x*_, which was prepared by adding lyophilized GO_*x*_/LO_*x*_ to APTMS (1 m, 3 μL) in PBS. The CFs were dipped into this solution for 1 h and then dried at 4 °C. The electrode APTMS/CF/Pd@Au/GO_*x*_ or APTMS/ CF/ Pd@Au/LO_*x*_ was prepared by further functionalizing the APTMS/CF system via growing Pd@Au through a sequential pathway. APTMS‐treated CFs were dipped in an ethanolic solution of HAuCl_4_ (10 mm) for 20 min and dried in an oven. The obtained CFs were soaked in 30% HCHO solution for 5 min and left in air for 10 min. The fibers turned dark reddish in color and were dried under vacuum. These CFs were then immersed in a PVP (1% solution)‐treated solution of Pd(acac)_2_ (15 mm) in DMF for 1 h and allowed to dry. Modified CFs were treated using EETMS solution (2 m); the reaction was allowed to occur at 40–50 °C. The obtained CFs were dried in vacuum. The protocol used for Pd@Au synthesis was reported in our previous studies.^[^ ^26^, ^29^, ^67^, ^68^, ^69^, ^70^, ^71^ ^]^ The Pd@Au‐functionalized CFs were dipped in a solution of GO_*x*_/LO_*x*_ [APTMS in PBS (3 μL)] for 60 min and allowed to dry at 4 °C. For the APTMS/CF/AOCE‐6 electrode, APTMS/CFs were treated with methanolic solution of AOCE – 6 (5 μL, 0.1 m) for 30 min and allowed to dry in an oven. The APTMS/CF/Pd@Au/AOCE‐6 electrode, APTMS/CF was treated as described above under APTMS/CF/Pd@Au/GO_*x*_ or APTMS/CF/Pd@Au/LO_*x*_ processing to give APTMS/CF/Pd@Au and subsequently treated with glutaraldehyde to generate aldehydic group‐terminated sites, followed by the steps for the APTMS/CF/AOCE‐6 electrode.

##### Preparation of gel Electrolyte with Microchannels

An agar agar/redox couple/antifouling agent gel electrolyte was prepared using the following procedure. 0.625 g of agar powder in PBS (0.1 m, 19 mL, pH 7.4) at 70 °C was prepared by constant stirring and kept for 15 min to completely dissolve the solute, resulting in a clear solution. Subsequently, potassium ferricyanide (0.5 m) was added dropwise into the agar solution and allowed to mix under stirring. Finally, the obtained slurry was poured into a container (with dimensions 50 mm × 15 mm × 15 mm in size) containing sections 5 × 15 × 15 mm in size, followed by the insertion of 3D‐printed microchannel plates in each unit. The slurry was allowed to cool down and solidify at room temperature. The slabs of gel electrolyte with casted microchannels were removed; CF WEs were introduced at the available slots.

##### Device Assembly

The ISF extraction and biomarker sensing device was designed using the approach of a medical syringe with a hypodermic needle. A 6 cm‐long syringe column was utilized and three rectangular orifices were made, which were nearly 10 mm below the top, each oriented 120° with respect to one another. The cap‐like structure with a MN array at the top was adhered on the syringe column using a cyanoacrylate glue. After the assembly dried, a thin and flat piece of cotton swab (dimensions similar to the inner diameter of the holder with a capillary‐like protrusion at the center) was inserted into the holder. The cotton swab (Figure S9, Supporting Information) was used as reservoir for ISF collection in this study (Figure 6). Similar sizes of thin cotton flat swabs were taken, and their dry weight (w_0_) was recorded. Later, they were weighed again after absorption; the weight (*w*_f_) reflects the sum of w_0_ and the weight of the fluid (w_l_) absorbed (w_0_ + w_l_). Therefore, the volume absorbed (*V*_l_) was calculated from the relation:

(5) $$V_{l}=\frac{\left(w_{f}-w_{0}\right)}{\rho }$$

where *ρ* is the density of fluid absorbed, which is taken as 1 g mL^−1^. The experiment was performed with same size swabs and the deviations were calculated (Table 1 and **3**).

**Table 3:** Record of volume collection

Time [min]	Weight of fluid (*w*_f_) Gms	*V*_l_ * = w*_l_/*ρ* [μL]
1	0.00235	5 E−5
5	0.0024	1 E−4
7.5	0.0027	4 E−4
10	0.0030	7 E−4
12.5	0.0038	0.0011
15	0.0042	0.0015
20	0.0047	0.0019
22.5	0.0052	0.0024
25	0.0058	0.0029

Subsequently, a slab of microchanneled gel electrolyte was placed to prevent reverse expulsion. Later, three replicas of three‐electrode configurations (combination of working/auxiliary/reference microelectrode assemblies with specific analyte sensors) were inserted through the orifices and sealed to avoid any air exchange. The assembled syringe‐type device was introduced with two additional features not seen in normal syringes: 1) a hook‐shaped clip, which is meant to lock the retracted plunger at required position, and 2) a compression spring, in which one end was fixed inside the syringe column (close to the top) while the other end was attached with the head of plunger. This approach helped to enhance the negative pressure convection. Therefore, with the help of hook at the back, the plunger can be held consistently for long periods. The pressure in the device was measured with a digital manometer. The WEs were prepared as follows: 1) functionalization of CFs in the aforementioned manner, 2) dried and trimmed CFs were adhered to copper foil substrates using conductive silver epoxy glue, and 3) the assembly was treated to obtain the required dimensions. Later, 0.5 mm‐thick, 1.5 cm‐long silver and platinum wires, which served as reference and auxiliary electrodes, along with each of the Wes, were configured to prepare three sets of three‐electrode systems. Each electrode set input was individually used to connect with the potentiostat circuit for electrochemical analysis.

##### Electrochemical Characterization and Performance of Biosensors In vitro

The electrochemical behavior of the modified and unmodified CF electrodes was evaluated using a DropSens SPELEC system (Metrohm AG, Herisau, Switzerland) via the cyclic voltammetric technique within a potential window of −0.3–0.6 V at a scan rate of 2 mV. Further, the sensitivity and selectivity of the WEs for glucose, lactate, and potassium ion sensing were analyzed using an amperometry scan. All of the WEs were incubated for 1000 s at certain fixed potentials to stabilize them before recording the analyte response. For glucose in vitro experiments, responses were recorded after 350 s with a physiological range of 0.075–350 μm using a potential step of −0.2 V over 1000 s. The electrochemical performance of the lactate biosensor toward lactic acid detection was evaluated over 1000 s at a stepping potential of −0.2 V with a concentration range of 0.01–75 μm. For potassium ions, responses were recorded with potassium chloride solutions ranging from 0.01 to 7.5 μm. Additionally, the apparent *K*_m_ was calculated in terms of diffusion. The general form of nonlinear reaction‐diffusion equations is given as

(6) $$\frac{\partial u(x,t)}{\partial t}=D\Delta^{2}u(x,t)+f(u(x,t))$$

This equation describes the density/concentration of substrate fluctuations in a material undergoing reaction‐diffusion. A reaction‐diffusion equation comprises a reaction term and a diffusion term. Δ^2^ denotes the Laplace operator.^[^ ^72^, ^73^, ^74^ ^]^ As such, the first term on the right‐hand side describes the diffusion, including *D* as diffusion coefficient. The second term, *f*(*u*), is a smooth function and describes the processes with real change. The substrate and product concentrations *S*(*t*, *x*) and *P*(*t*, *x*) are functions of two variables, the distance to the biosensor electrode *x* and time *t*. The substrate and product concentrations *S*(*t*, *x*) and *P*(*t*, *x*) are functions of two variables, the distance to the biosensor^[^ ^75^, ^76^ ^]^ electrode *x* and time *t*. The values 0 < *x* < *a*_e_ correspond to the enzyme‐containing layer, which corresponds to the points inside the outer membrane.^[^ ^77^ ^]^ For 0 < *x* < *a*_e_ and *t* > 0, the dynamics of substrate and product concentrations are described by the following nonlinear reaction‐diffusion equations.

(7) ∂S∂t=∂(DS(x)∂S∂x)∂x−α(x)VmaxSKm+S

(8) ∂P∂t=∂(DP(x)∂P∂x)∂x−α(x)VmaxSKm+S

where the functions *D*_S_(*x*), *D*_P_ (*x*), and *α*(*x*) are defined as follows.

(9) $$D_{S}(x)=\{D_{\text{Se}}=0<x\leq a_{e}$$

(10) $$D_{P}(x)=\{D_{\text{Pe}}=0<x\leq a_{e}$$

*D*_Se_ and *D*_Pe_ are the substrate and product diffusion coefficients in the enzyme‐containing electrode and diffusion layer. It was assumed that the concentration of substrate in the solution (*S*_0_) remains constant during the entire process time. At the beginning of process (*t* = 0), there is neither substrate nor product inside the enzyme‐containing layer and outer surface. As a response of electrochemical biosensor, the steady‐state current density (*I*) is used.

(11) $$I(t)=n_{e}F\;D_{\text{Pe}}\frac{\partial P}{\partial x}$$

where ne is the number of electrons involved in the charge transfer at the electrode surface, and *F* is the Faraday constant. The diffusion module allows one to compare the rate of the enzyme reaction (Figure 5), *V*_max_/*K*_m_, with mass transport through the enzyme‐containing layer by the relation

(12) $$\sigma^{2}=\frac{V_{\max}a_{e}^{2}}{K_{m}D_{\text{Se}}}$$

The value of diffusion module varied with respect to *a*_e_ and diffusion coefficients, and apparent *K*_m_ was calculated using *a*_e_: 2 mm, *D*_Se_: 3.5 μm^2^ s^−1^, and *σ*: 0.9782.

##### Ex vivo ISF Extraction and Testing of Biomarkers in Biofluid

The oblique design of an individual needle in an MN array (shown in Figure 3) combined with pressure‐driven drag facilitated the successful withdrawal of ISF from porcine skin. The MN^[^ ^78^, ^79^ ^]^‐attached suction holder was pressed against the fresh porcine skin for ≈8–10 min. Similar to a disposable medical syringe (5 mL), a low‐pressure area of −74 kPa (measured with digital manometer) was created through the suction setup (shown in Figure S10, Supporting Information). A hook‐shaped clip was attached toward the back to lock the retracted plunger so that it could be held consistently for long and serve the purpose. After stopping the outward perpendicular force, clear ISF was drawn out from the pierced holes. Through the capillary action, ISF was absorbed into the cotton patch, transferred to the microchanneled gel electrolyte, and ultimately brought to the WE surfaces. The ISF from the punctured skin segment was quantified as ≈3.0 μL (per 4 × 4 set of MNs). The ISF samples obtained from porcine skin were tested for ex vivo electrochemical measurements of glucose, lactate, and potassium ion levels. The CV responses from these fluids were matched with those of in vitro results to determine the approximate concentration of biomarkers present in the fluid.

##### Geometry and Numerical Assumptions

A microelectrochemical cell working system was considered to explore the diffusion or transport of analytes on the CF WEs. The 3D geometric model in the form of a cylinder with a radius 1 mm and a height of 0.5 mm was made to mimic the experimental cell design with one WE on its surface. For computational evaluations, a simple environment of 2D simulation domain (90° sector) was selected. The dimensions of this 2D frame included the radius (*r*_c_) of the cylinder and a square electrode region with sides *r*_e_. As shown in Figure 10B, the electrode area domain was meshed using a free triangular type with an extremely fine element size. While the remaining portion was mapped with an extra fine mesh type to ensure that convergence was reached. In the cell surrounding, analyte transport was governed by capillary action, diffusion, and convection created in the system. The conversion of diffused analytes followed a fast irreversible transformation reaction. The time‐dependent mass transport was computed using the equations

(13) $$\frac{\partial c_{i}}{\partial t}+\nabla *J_{i}+v*\nabla c_{i}=R_{i}$$

(14) $$J_{i}=-D_{i}\nabla c_{i}$$

where *C*_i_ represents the analyte concentration in the sample (μmol m^−3^), Ji represents the flux of the analyte, *R*_*i*_ represents the mass source of analyte, *D*_i_ represents the compound diffusion coefficient in the sample (10^−9^ m^2^ s^−1^), and *u* represents the sample velocity field (m s^−1^).

(15) $$-n*J_{i}=v_{i}r_{e}$$

(16) $$r_{e}:c_{\text{lim}}=0$$

(17) $$\sum_{i,v_{i}<0}v_{i}S_{i}\to \sum_{i,v_{i>0}}v_{i}S_{i}$$

Equation (13) and (14) are Nernst–Planck–Poisson equations for the nonlinear distribution of the sample. Equation (15)–(17) assume a time‐dependent fast irreversible reaction in the region *r*_e_^2^ (electrode area).

## Conflict of Interest

The authors declare no conflict of interest.

## Supporting information

Supplementary Material

## Data Availability

The data that support the findings of this study are available from the corresponding author upon reasonable request.
