# Supplementary material for: Microneedle‐Integrated Device for Transdermal Sampling and Analyses of Targeted Biomarkers
Source: Small Sci. 2023 Apr 22;3(6):2200087. doi: 10.1002/smsc.202200087 (PMC11936029; doi:10.1002/smsc.202200087)
Supplement: Supplementary file 1 — Supplementary Material [file SMSC-3-2200087-s001.pdf]

Supporting Information

Microneedle Integrated Device for Transdermal Sampling and Analyses of Targeted Biomarkers

Shubhangi Shukla[1], Sina Azizi Machekposhti[1], Naveen Joshi[2], Pratik Joshi[2], Roger J Narayan[1*]

[1] Joint Department of Biomedical Engineering, North Carolina State University, Raleigh, NC 27695-7907, USA

[2] Department of Materials Science and Engineering, North Carolina State University, Raleigh, NC 27695-7907, USA

*Corresponding Author E-mail: rjnaraya@ncsu.edu (Roger Narayan)

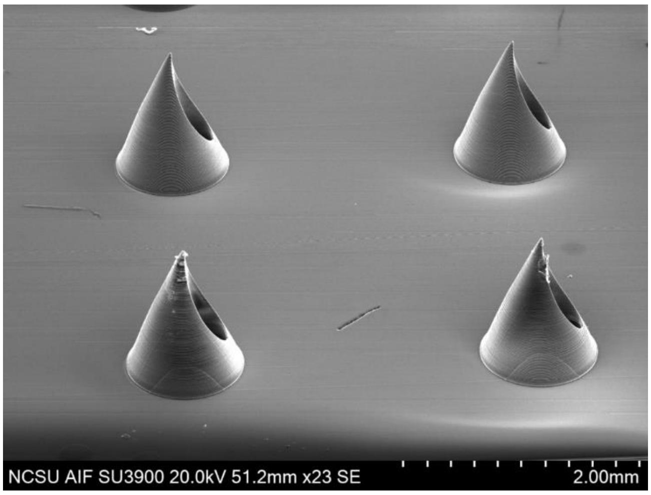

Figure S1. SEM micrographs showing the cross-sectional view of the MN array.

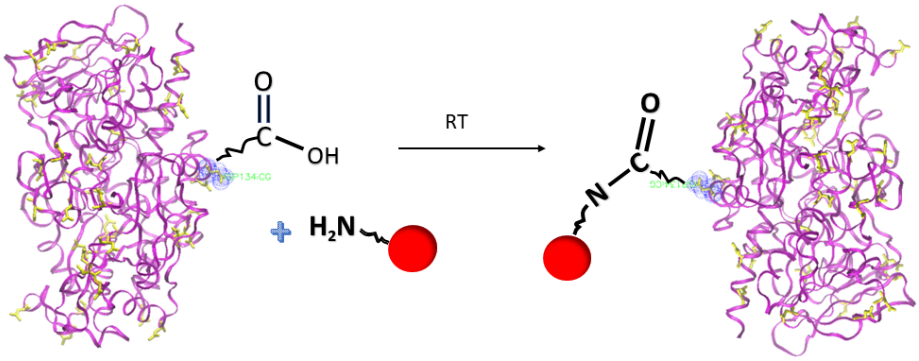

Figure S2. Possible mechanism for enzyme immobilization.

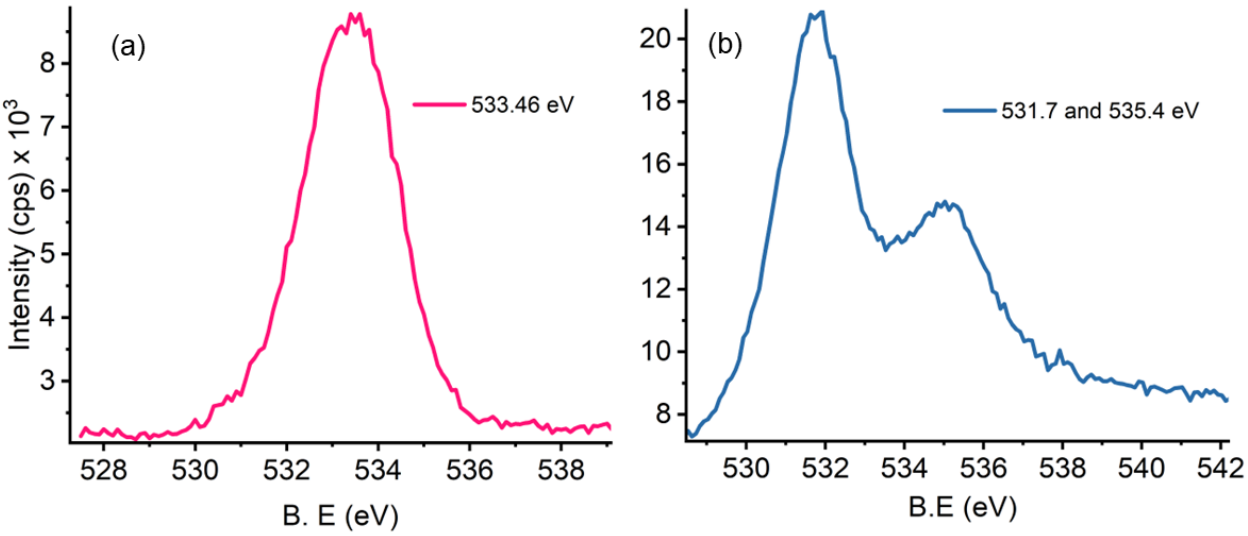

Figure S3. XPS O 1s spectra of a) unmodified CF, b) CF/APTMS/Pd@Au/AOCE-6, and c) CF/APTMS/Pd@Au/LOx

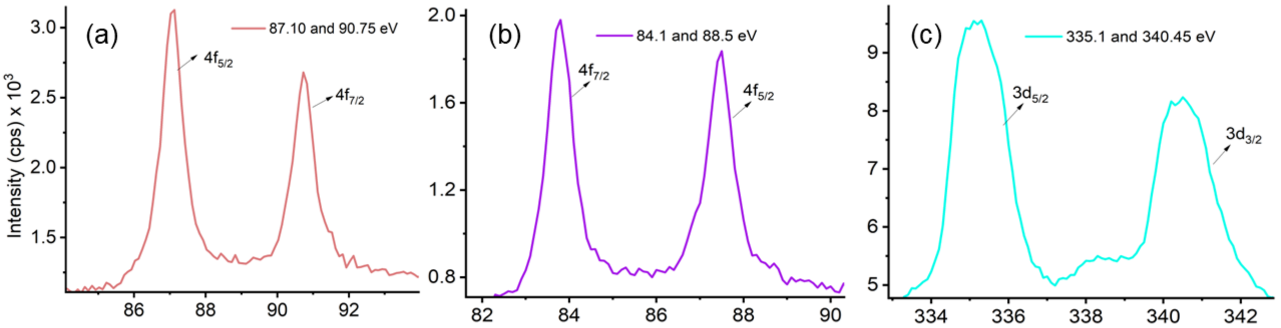
(a)
87.10 and 90.75 eV
$4f_{5/2}$
$4f_{7/2}$
Intensity (cps) x 10^3
B.E. (eV)
(b)
84.1 and 88.5 eV
$4f_{7/2}$
$4f_{5/2}$
B. E. (eV)
(c)
335.1 and 340.45 eV
$3d_{5/2}$
$3d_{3/2}$
B.E. (eV)

Figure S4. XPS profile for (a) Au in CF/APTMS/AuNPs, (b) Au in CF/APTMS/Pd@AuNPs and (c) Pd in CF/APTMS/Pd@AuNPs.

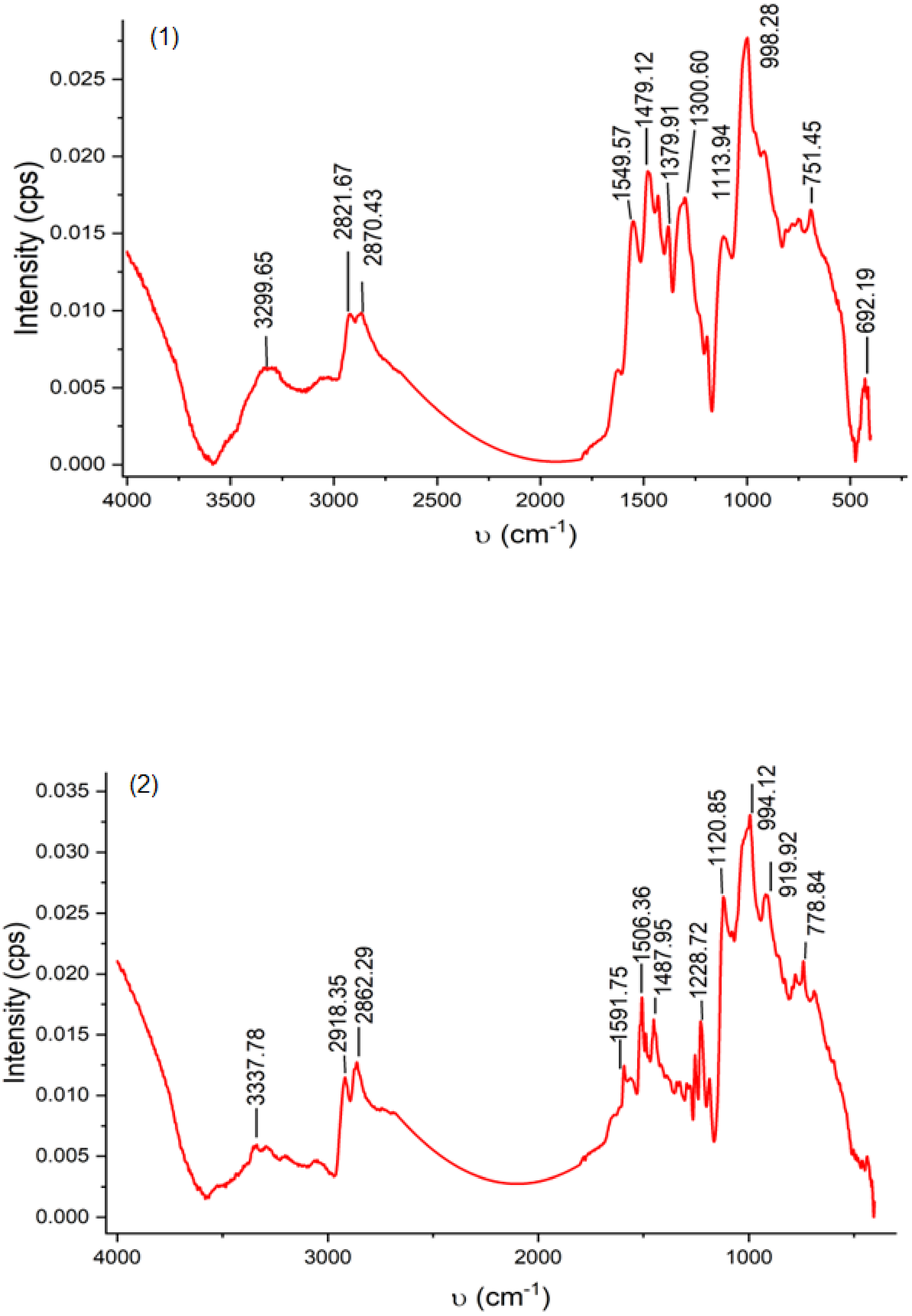

Figure S5A. Peak indexing of FTIR profiles for (1) CF/APTMS/Pd@Au/LOx and (2) CF/APTMS/Pd@Au/GOx.

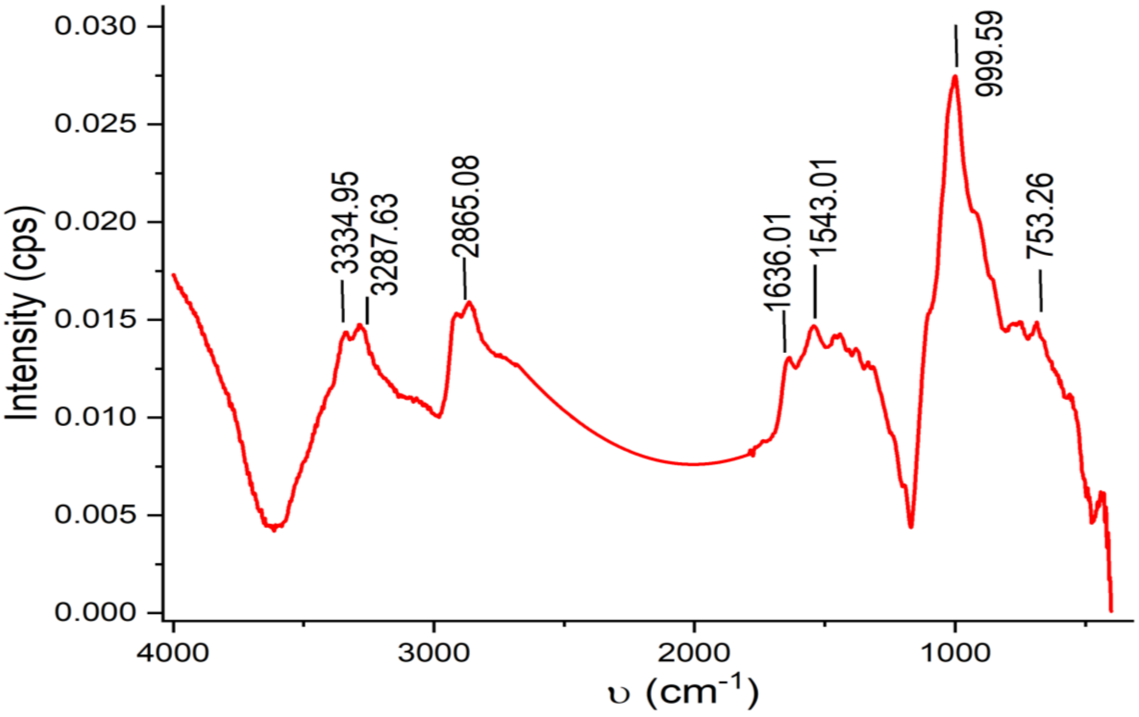

Figure S5B. Peak indexing of FTIR profile for CF/APTMS/Pd@Au/AOCE-6.

Table S1a. Elemental analysis of the surface of the carbon fibers in CF/APTMS.

Quantification

	BE [eV]	FWHM [eV]	RSF	Atomic conc. [%]	Error [%]	Mass conc. [%]	Error [%]
O 1s	533.45	0.00	0.58	16.3	0.13	20.5	0.16
C 1s	285.55	0.00	0.28	80.0	0.13	69.5	0.16
Si 2p	102.20	0.00	0.20	3.7	0.15	10.0	0.40

Table S1b. Elemental analysis of the surface of the carbon fibers in CF/APTMS/AuNPs.

Quantification

	BE [eV]	FWHM [eV]	RSF	Atomic conc. [%]	Error [%]	Mass conc. [%]	Error [%]
N 1s	400.20	0.00	0.48	9.6	0.35	8.9	0.33
Cl 2p	197.20	0.00	0.89	0.6	0.12	1.3	0.28
Si 2p	102.20	0.00	0.33	9.8	0.25	18.1	0.43
O 1s	532.05	0.00	0.78	24.0	0.15	25.4	0.20
Au 4f	83.45	0.00	6.25	0.1	0.00	1.9	0.03
C 1s	284.95	0.00	0.28	55.9	0.30	44.3	0.32

Table S1c. Elemental analysis of the surface of the carbon fibers in CF/APTMS/Pd@Au/enzyme.

Quantification

	BE [eV]	FWHM [eV]	RSF	Atomic conc. [%]	Error [%]	Mass conc. [%]	Error [%]
Pd 3d	339.03	0.00	5.36	5.0	0.06	30.8	0.24
O 1s	531.93	0.00	0.68	46.5	0.28	30.2	0.26
Au 4f	87.13	0.00	6.28	0.4	0.01	4.0	0.06
C 1s	284.83	0.00	0.28	51.4	0.26	30.0	0.18
Si 2p	102.20	0.00	0.20	3.0	0.25	4.0	0.43

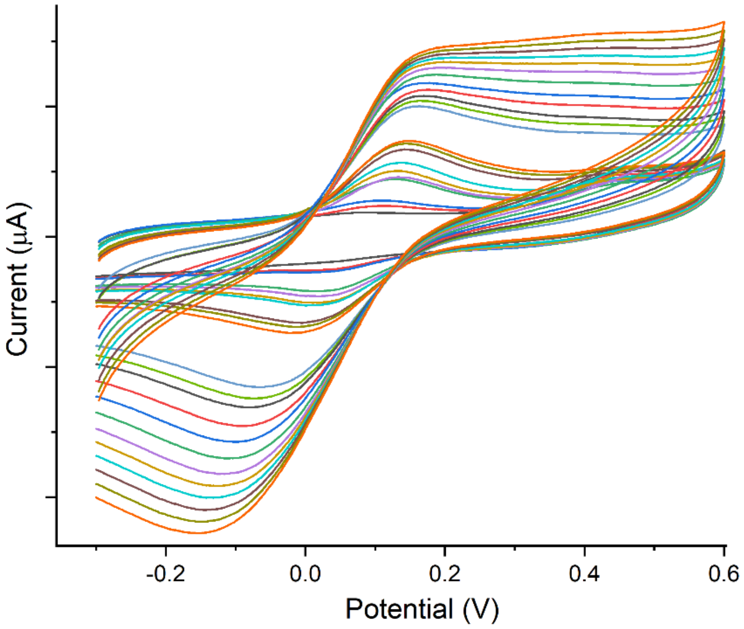

Figure S6. Optimization of CV voltammogram for potassium ions on CF-WE_3.

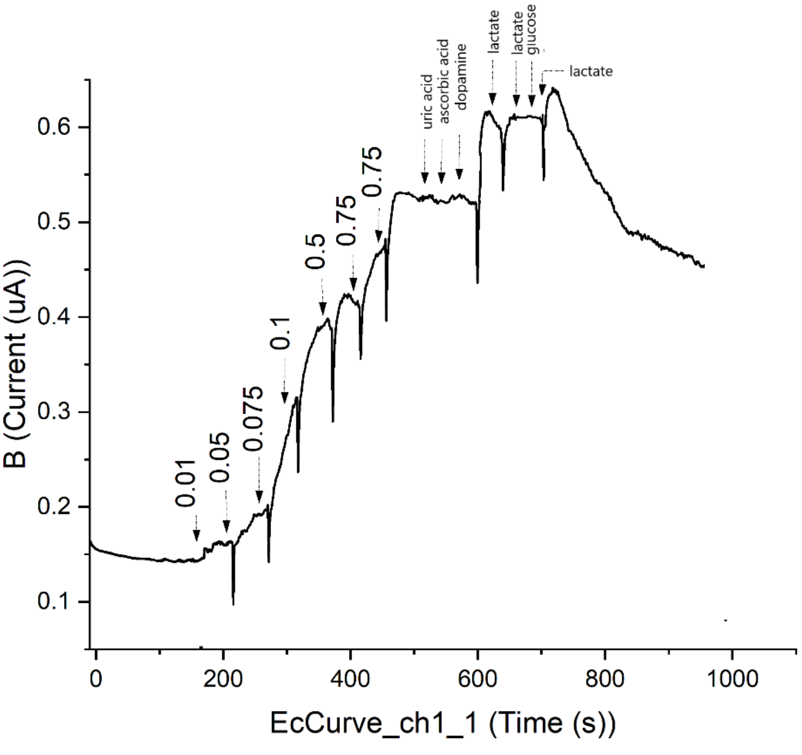

Figure S7. Amperometric response of WE1 to different concentrations of lactate (0.005 to 0.75 μM) and selectivity in the presence of uric acid, ascorbic acid, dopamine, and glucose.

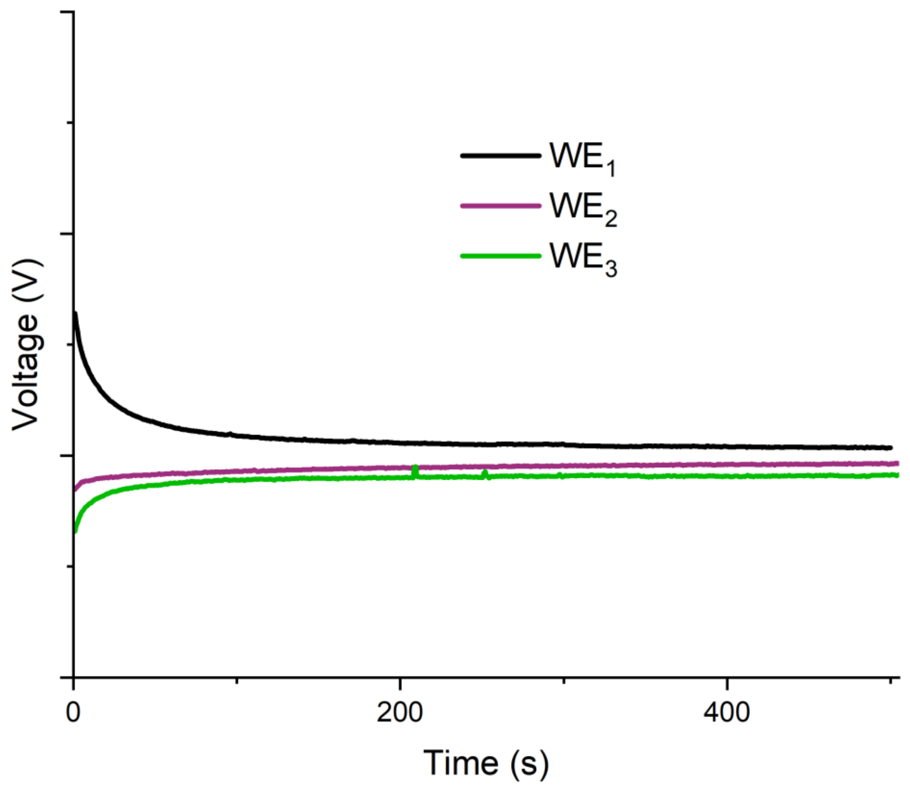

Figure S8. Cross talk study for WE1, WE2, and WE3 electrodes.

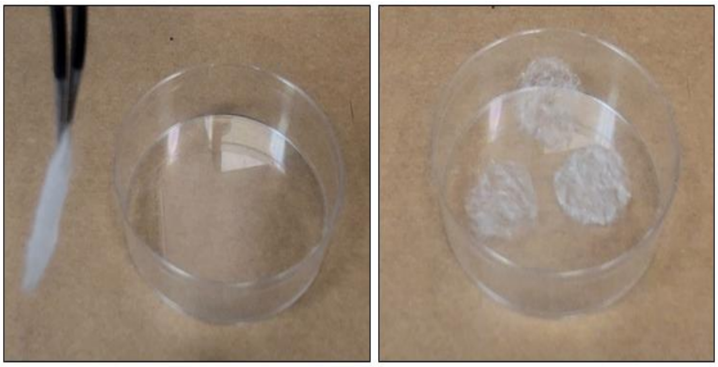

Figure S9. Flat cotton swabs

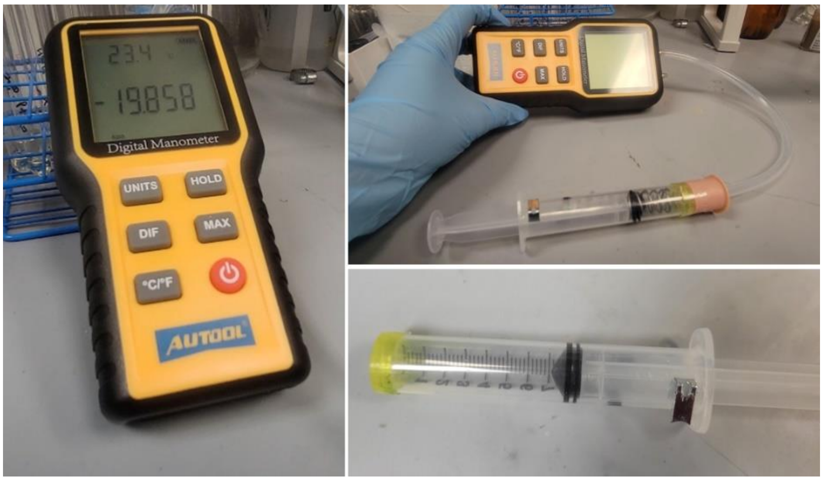

Figure S10. The digital manometer for pressure measurement in device.
